# A scoping review of local quality improvement using data from UK perioperative National Clinical Audits

**DOI:** 10.1186/s13741-022-00273-0

**Published:** 2022-08-29

**Authors:** Duncan Wagstaff, Samantha Warnakulasuriya, Georgina Singleton, Suneetha Ramani Moonesinghe, Naomi Fulop, Cecilia Vindrola-Padros

**Affiliations:** 1 Department of Anaesthesia, University College London Hospital, London, UK; 2 PQIP, National Institute of Academic Anaesthesia Health Services Research Centre, Royal College of Anaesthetists, London, UK; 3 Division of Surgery and Targeted Intervention, University College London, London, UK; 4 Department of Applied Health Research, University College London, London, UK

**Keywords:** Quality improvement, Clinical audit, Perioperative medicine, Anaesthesia, Surgery

## Abstract

**Background:**

Significant resources are invested in the UK to collect data for National Clinical Audits (NCAs), but it is unclear whether and how they facilitate local quality improvement (QI). The perioperative setting is a unique context for QI due to its multidisciplinary nature and history of measurement. It is unclear which NCAs evaluate perioperative care, to what extent their data have been used for QI, and which factors influence this usage.

**Methods:**

NCAs were identified from the directories held by Healthcare Quality Improvement Partnership (HQIP), Scottish Healthcare Audits and the Welsh National Clinical Audit and Outcome Review Advisory Committee. QI reports were identified by the following: systematically searching MEDLINE, CINAHL Plus, Web of Science, Embase, Google Scholar and HMIC up to December 2019, hand-searching grey literature and consulting relevant stakeholders. We charted features describing both the NCAs and the QI reports and summarised quantitative data using descriptive statistics and qualitative themes using framework analysis.

**Results:**

We identified 36 perioperative NCAs in the UK and 209 reports of local QI which used data from 19 (73%) of these NCAs. Six (17%) NCAs contributed 185 (89%) of these reports. Only one NCA had a registry of local QI projects. The QI reports were mostly brief, unstructured, often published by NCAs themselves and likely subject to significant reporting bias. Factors reported to influence local QI included the following: perceived data validity, measurement of clinical processes as well as outcomes, timely feedback, financial incentives, sharing of best practice, local improvement capabilities and time constraints of clinicians.

**Conclusions:**

There is limited *public reporting* of UK perioperative NCA data for local QI, despite evidence of improvement of most NCA metrics at the national level. It is therefore unclear *how* these improvements are being made, and it is likely that opportunities are being missed to share learning between local sites. We make recommendations for how NCAs could better support the conduct, evaluation and reporting of local QI and suggest topics which future research should investigate.

**Trial registration:**

The review was registered with the International Prospective Register of Systematic Reviews (PROSPERO: CRD42018092993).

**Supplementary Information:**

The online version contains supplementary material available at 10.1186/s13741-022-00273-0.

## Background

Local quality improvement (QI) initiatives, including audit and feedback, have the potential to substantially improve healthcare services, but implementation can be variable and is not always sustainable (Ivers et al. 2012). A common barrier to successful QI is the design and use of data monitoring systems (Dixon-woods et al. 2012). Difficulties with the effective use of data include the following: defining appropriate quality metrics (Lilford et al. 2004; Greenhalgh et al. 2017); collecting data efficiently; feeding back results in a timely, meaningful and accessible fashion (Ivers and Barrett 2018); and lack of skills to translate these data into effective organisational responses (Ross et al. 2016). Reporting data may also have unintended consequences including gaming, distortion of healthcare systems to fit measurement systems, data overload and excessive burdens of data collection on clinical staff (Shah et al. 2018).

National and regional measurement programmes in North America have demonstrated mixed success in using routinely collected data to support local QI (Etzioni et al. 2015; Montroy et al. 2016; Vu et al. 2018). US hospitals which pay to contribute to QI programmes often have better outcomes than those that do not. The UK pursues a mandatory approach to clinical data collection in the NHS via National Clinical Audits (NCAs). Approximately, 160 NCAs operate across or within the four devolved nations. English NCAs are overseen by the Healthcare Quality Improvement Partnership (HQIP) and provided by either HQIP itself, NHS organisation or Royal Colleges. Wales participates in most of these programmes, and Scottish NCAs are coordinated by Information Services Directorate Scotland. NCAs vary according to factors including the following: whether they report data at the level of local units or individual clinicians, whether they publicly report local data and the frequency, timeliness and nature of local feedback.

The emphasis of NCA measurement has historically been for *quality assurance* but is now broadening to also promote *quality improvement *(Sinha 2016; Peden and Moonesinghe 2016)*. *NCAs can support improvement in multiple ways, including the following: by using national level data to drive improvement at that level (Vallance et al. 2018; Neuburger et al. 2015); by identifying and contacting units which are deemed ‘negative outliers’, by providing data for secondary use by programmes seeking to reduce national variation (such as the Getting It Right First Time programme), or by feeding back local data directly to all participating units to support local QI according to local needs and circumstances. However, issues around data quality, relevance of feedback, reach within healthcare settings and clinician engagement have meant that their full potential to support local improvement has not always been fulfilled (Taylor et al. 2016; Allwood 2014; RCP, HQIP 2018; Sykes et al. 2020). National institutions in the UK are now prioritising the optimisation of this practice (Foy 2017).

Previous studies have suggested that QI is often poorly reported in healthcare literature because of divergent understandings of QI, difficulties describing interventions/contexts or structural barriers impeding the reporting of QI in the existing biomedical research publishing infrastructure (Jones et al. 2019). There are diverse repositories for sharing QI, but those that do exist vary significantly in their methodology, and few include evaluations of their impact (Bytautas et al. 2017). Consequently, QI is most often reported in the grey literature, often using unstructured formats and without formal peer review. The quality of QI reports which do get published has been criticised as lacking key details such as those necessary to replicate the intervention(s) being described (Levy et al. 2013; Jones et al. 2016). Furthermore, there is a tendency to only report those projects which achieve ‘positive’ results, leading to significant publication bias (Taylor et al. 2014).

Perioperative care encompasses the period before, during and after surgery. The UK perioperative context is a unique environment for measuring and improving quality as it increasingly considers issues across all surgical specialties, i.e. as a *sector *(The King's Fund 2018). The discrete nature of most perioperative encounters lends itself to measurement. Perioperative quality has been an emotive issue due to well-publicised examples of poor practice (The Bristol Royal Infirmary Inquiry 2001). Policy-makers have responded with quality assurance measures including numerous NCAs with disparate methodologies including how they feed back data and whether they encompass a whole surgical specialty, a particular procedure or wider perioperative care. There is no definitive list of ‘perioperative’ NCAs in the UK.

This scoping review therefore seeks to identify and characterise NCAs which evaluate perioperative care in the UK, map publicly available reports of the use of data from these NCAs for local QI projects and identify factors reported to influence this use. We go on to make recommendations for how NCAs might best support local QI going forward.

## Methods

### Design

The review was registered with PROSPERO, an international database of prospectively registered reviews (reference CRD42018092993).

A scoping review methodology was deemed most appropriate to describe this heterogeneous literature and achieve our aims. Scoping reviews are used to explore the extent of existing research activity, map any gaps in the literature and inform policy, practice and research (Tricco et al. 2016; Levac et al. 2010). We chose to use the framework as suggested by Arksey and O-Malley (Arksey and O’Malley 2005), further developed by both Levac and Daudt (Levac et al. 2010; Daudt et al. 2013). This manuscript follows the Preferred Reporting Items for Systematic Reviews and Meta-Analyses extension for Scoping Reviews (PRISMA-ScR) checklist.

We have used the following definitions:

- *Perioperative*: ‘from the moment the decision to undergo surgery has been taken until the patient has returned to best health and no longer requires specialist input’ (The Royal College of Anaesthetists 2015)
- *Quality Improvement*: ‘the use of systematic methods and tools to improve outcomes for patients on a continuous basis’ (Robert et al. 2011)
- *Local QI*: ‘QI occurring within all/part of the organisation(s) delivering direct patient care’ (HQIP 2017)

A two-phase approach has been adopted for this study:

- Phase 1: identify and characterise a list of perioperative NCAs in England, Wales and Scotland.
- Phase 2: search for, and review, evidence of data from those perioperative NCAs being used for local QI

### Phase 1: identification and characterisation of perioperative NCAs

#### Identifying perioperative NCAs

Two reviewers with expertise in the perioperative setting hand-searched the Healthcare Quality Improvement Partnership (HQIP) directory — the coordinator of NCAs in England and Wales, and the list of Scottish Healthcare Audits (SHA). The first search in November 2017 was updated in January 2020. Audits were selected for inclusion as follows:

1. Reported data on perioperative care
2. Had released at least two reports or set of results (so that QI has had an opportunity to take place)
3. Reported provider-level data
4. Were either of the following:
  a Included in the NHS England Quality Account reporting requirements OR
  b Included in the list of Scottish Healthcare Audits run by Information Services Directorate Scotland OR
  c Included in the list of National Audits mandated by the Welsh National Clinical Audit and Outcome Review Advisory Committee (NCAORAC)

We excluded confidential enquiries or outcome programmes because these national projects investigate individual incidents or selected themes rather than auditing patient care.

#### Characterising perioperative NCAs

Evaluations of NCAs were conducted by HQIP in 2014, and Scottish Healthcare Audits in 2015, with NCAs completing questionnaires describing their design, conduct and impact (Phekoo et al. 2014; Baird 2015). In 2018, this process was refreshed by HQIP with the Understanding Practice in Clinical Audit (UPCARE) tool. These self-assessments of NCAs were examined (where available) along with other public material in order to describe their structures, processes and impacts. These findings have been analysed using categories drawn from the three self-assessment programmes, as well as from relevant literature synthesising how audit can support improvement (Ivers et al. 2012; Ivers et al. 2014; Dixon 2013).

### Phase 2: studies which describe the use of perioperative NCA data for local QI

#### Searching for QI reports

A systematic search (online Supporting information Fig. S1) of the literature was performed using the names of perioperative NCAs and QI. Subject headings, keywords and synonyms were used where appropriate. This search was performed using the following databases: MEDLINE, CINAHL Plus, Web of Science, Embase, Google Scholar and HMIC (Health Management Information Consortium). The review was conducted in January 2018 and limited to English language manuscripts published up to December 2017. An update was performed in January 2020 to include manuscripts published between January 2018 and December 2019. All types of manuscripts and studies were deemed eligible for inclusion.

The following additional sources were also hand-searched (during both the initial search and the update): bibliographies of included articles; reports and websites of each perioperative NCA; supplements containing conference proceedings from the *Anaesthesia* and *British Journal of Surgery*; the HQIP website; websites of the Health Foundation, King’s Fund and Nuffield Trust; archives of *BMJ Open Quality* and *BMJ Quality & Safety*. We then performed citation searching where possible using the ‘cited reference search’ function in the Web of Science platform.

#### Stakeholder consultation

We consulted HQIP and twice contacted the NCA providers for examples of local QI. Preliminary results of this scoping review were presented to an independent panel of perioperative clinicians, academics, representatives of the RCOA and lay members in May 2018. Feedback from these consultations did not identify any gaps in our search strategy. Their comments have been included in our analysis.

#### Screening of QI reports

The search results were imported into Mendeley (© 2018 Elsevier B.V.). Duplicates were removed, titles were screened by one reviewer and then abstracts were screened independently by two reviewers. Discrepancies between these reviewers (involving five reports) were resolved after discussion. One reviewer reviewed all of the full-text reports, and a second reviewer independently reviewed a random sample of 10% of all manuscript types. No discrepancies were found between reviewers at this stage.

#### Data charting

A charting template (online Supporting information Fig. S2) was created in Research Electronic Data Capture platform (REDCap 7.4.9—© 2018 Vanderbilt University) and iteratively refined during review of the reports. This form was based upon previous research investigating how audit data had been used for improvement (Taylor et al. 2016; Benn et al. 2015) and contained fields describing the manuscript format and target audience; ‘how’ NCA data were used for improvement and what the impact was. One reviewer performed data charting for all the articles, and a second reviewer cross-checked a random sample of 10% of articles within each manuscript category, finding no discrepancies.

#### Data synthesis

Quantitative data were summarised using descriptive statistics. Qualitative data were coded and synthesised using framework analysis (Gale et al. 2013). The framework included deductive categories described above (online Supporting information Fig. S2) and those arising inductively from the data. The findings are presented below as a narrative review.

#### Quality assessment

QI reports, or empirical evaluations thereof, published in scholarly literature were assessed according to the Standards for Quality Improvement Reporting Excellence (SQUIRE) 2.0 guidelines published in 2015 (Ogrinc et al. 2016).

#### Patient and public involvement

We are grateful for feedback on an early draft of our findings from lay members of the Royal College of Anaesthetists.

## Results

### Phase 1

#### Identification of perioperative NCAs

The reviewers identified 36 perioperative NCAs for inclusion (online Supporting information Fig. S3 & Table S1), of which 31 were based in England and Wales and five operate in Scotland. The reviewers erred on being inclusive when they struggled to decide whether an NCA was ‘perioperative’ or not, for example by including intensive care NCAs.

#### Characterisation of perioperative NCAs

Self-assessments were available for 21 (58%) of the 36 NCAs (online Supporting information Tables S2 and S3).

#### NCA structures

The median start time of the NCAs was 2011 (range 1994–2017). Surgical specialties and Royal Colleges constituted the largest group of perioperative NCA providers in England and Wales (48%). NCAs measured different aspects of care: 13 (33%) NCAs focussed on a *particular medical condition* (e.g. National Prostate Cancer Audit), 12 (33%) NCAs focussed on *specific procedure* (e.g. National Joint Registry), 6 (17%) NCAs audited a *whole specialty* (e.g. Adult Cardiac Surgery) and 5 (14%) NCAs discussed aspects of *perioperative care* (e.g. PQIP). All NCAs analysed performance at the level of hospitals, but 16 (44%) also published outcomes of individual surgeons. Nineteen (53%) NCAs described taking a QI approach in their protocol or website. The most commonly stated purpose of NCAs was to reduce clinical variation at the national level.

#### NCA processes

Only 8/21 (38%) of the NCAs with self-assessments reported making real-time data available to providers. Education sessions were the most commonly employed intervention to support local improvement and have been used by 16 (44%) of NCAs. Clinical recommendations were universally made at a national level, but no evidence was found of an NCA providing individualised action plans for hospitals to make improvements. Financial incentives, in the format of best practice tariffs, have been used by five (14%) NCAs: the NHS patient-reported outcome measures (PROMs) audit, the National Hip Fracture Database (NHFD), the National Joint Registry (NJR), the Trauma Audit and Research Network audit (TARN) and recently the National Emergency Laparotomy Audit (NELA). One NCA (NHFD) had an accessible registry of local QI projects using their data. Four NCAs (TARN, STAG, SICSAG and NELA) reported awarding prizes to projects performing local QI with their data.

#### Self-reported improvement using NCA data

At the national level, 97% of process/outcome measures for which longitudinal data were available demonstrated improvement in their most recent reports. A total of 16/21 (76%) NCAs self-assessed that their data had been used for local QI.

### Phase 2

#### Selecting QI reports

**Fig. 1:**
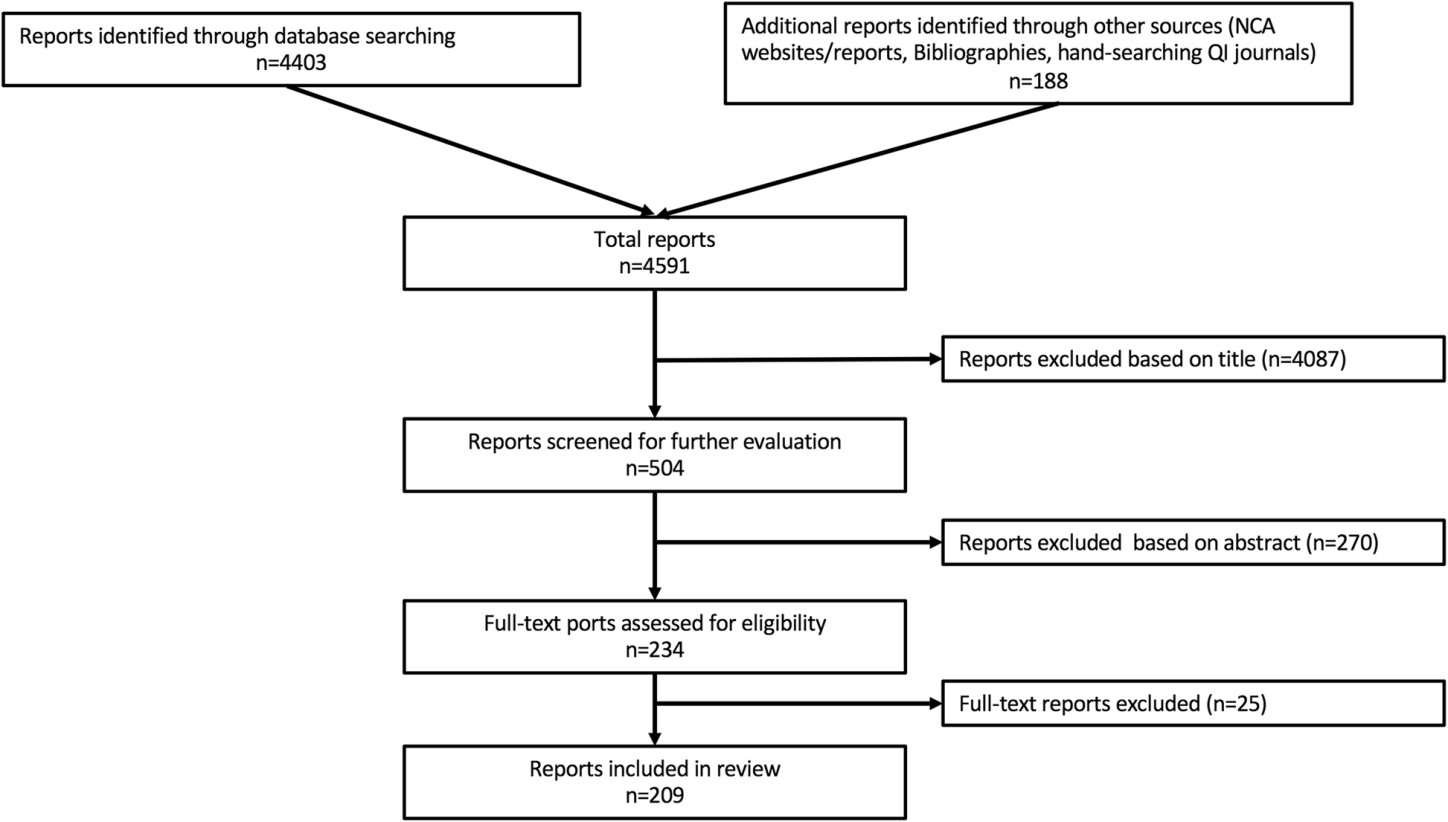
Flowchart showing selection of QI reports for inclusion

Two-hundred nine reports of local QI using perioperative NCA data were identified (Fig. 1 and online Supporting information Table S4).

#### Data from which NCAs have been used for local QI?

Evidence of local QI was found for 19 (73%) NCAs. Six (17%) NCAs collectively contributed 185 (89%) of all reports (Table 1). Reporting of local QI does not appear to be associated with the duration of the NCA (online Supporting information Fig. S4).

**Table 1:** The perioperative NCAs whose data was used in QI reports

Acronym	Full name	Duration of NCA (years)	No. of QI reports	% of total QI reports
NHFD	National Hip Fracture Database	13	67	31
NELA	National Emergency Laparotomy Audit	8	63	29
STAG	Scottish Trauma Audit Group	9	19	9
NLCA	National Lung Cancer Audit	15	16	7
PROMs	Elective Surgery — National PROMs Programme	11	10	5
TARN	Major Trauma Audit	19	10	5
PQIP	Perioperative Quality Improvement Programme	3	5	2
SHFA	Scottish Hip Fracture Audit	19	5	2
ICNARC-CMP	Intensive Care National Audit and Research Centre-Case Mix Programme	26	3	1
SICSAG	Scottish Intensive Care Society Audit Group	8	3	1
SSIS	Surgical Site Infection Surveillance Service	25	3	1
NJR	National Joint Registry	18	2	1
NVR	National Vascular Registry	8	2	1
DAHNO	Head and Neck Cancer Audit	17	1	0.5
NBOCAP	National Bowel Cancer Audit	17	1	0.5
NOGCA	National Oesophago-Gastric Cancer Audit	9	1	0.5
PICANet	Paediatric Intensive Care Audit Network	16	1	0.5
NPCA	National Prostate Cancer Audit	6	1	0.5
ACS	Adult Cardiac Surgery	23	1	0.5
**Totals**			**214**	**100**

#### Characteristics of QI reports

**Fig. 2:**
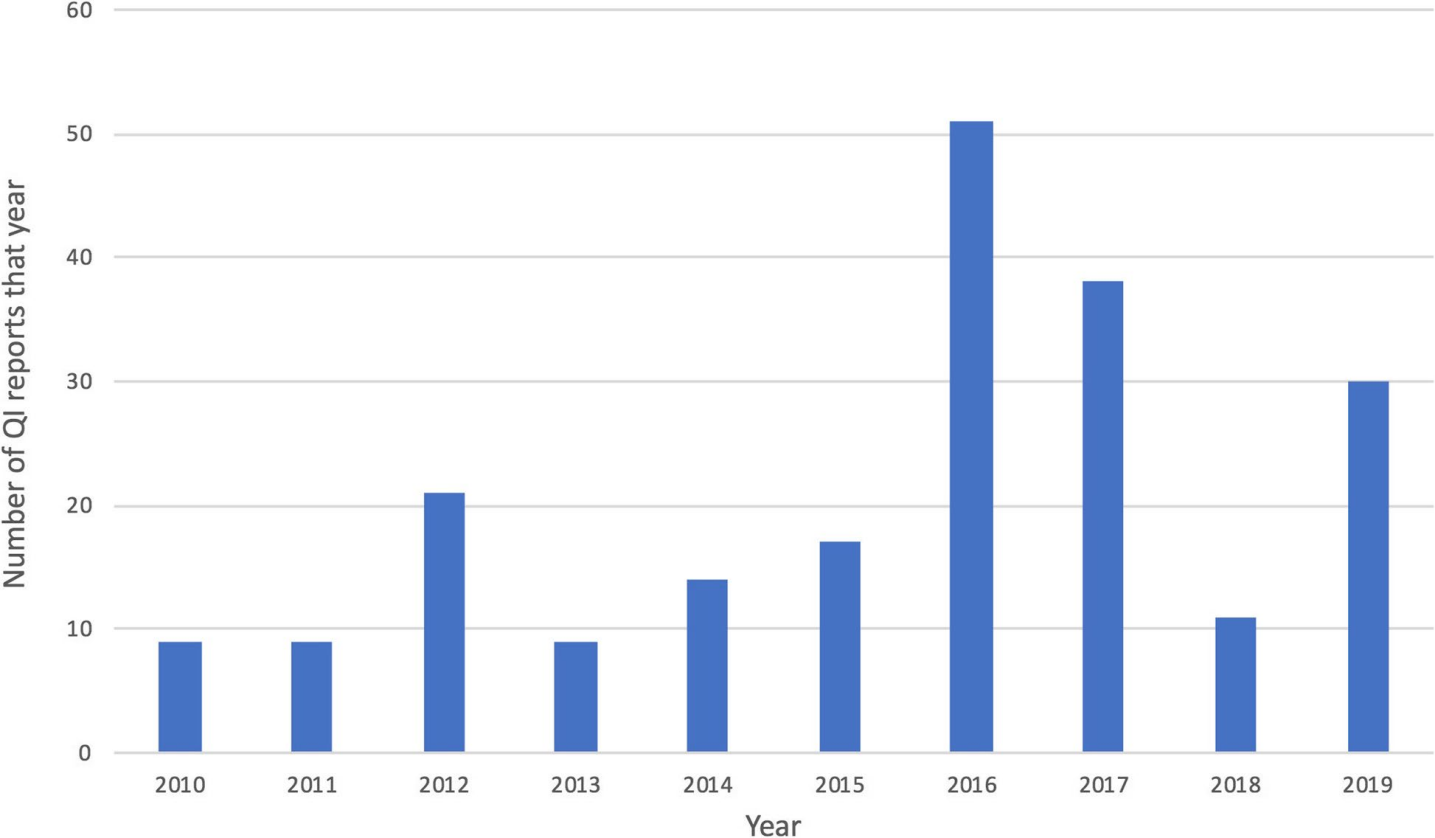
Frequency of study publication by year

The majority of reports (64%) were unstructured vignettes published within NCA annual reports or on their websites (online Supporting information S5). No reports were found dating from before 2010. The increasing reporting of local QI since then (Fig. 2) was largely driven by publication of vignettes in NCA annual reports, and abstract/poster competitions, particularly by the two NCAs whose data was most commonly used (National Hip Fracture Database and the National Emergency Laparotomy Audit).

Process indicators were the most common type of indicator to be used for QI projects (69% of reports), compared to 45% of reports using outcome indicators (online Supporting information Table S6). Six QI reports (3%) used data from the 16 (44%) NCAs which reported outcomes of individual clinicians. Data were most commonly used to monitor ongoing QI projects or to identify targets for new projects (online Supporting information Table S7).

#### Impact(s) of the reported local QI

One-hundred thirty-seven (66%) reports explicitly reported the impact of the QI project (online Supporting information Table S8). Where impacts were reported, 93% were positive. The benefits of QI projects beyond the primary indicator were stated by 61 (29%) reports and included the following: building QI and data skills and capacity, building other skills (e.g. leadership and communication), improving team-working, reduction in clinical hierarchies, improving processes and skills for sharing and using data and improving information to share with patients. Harms which were reported to be associated with QI projects were the displacement of previous improvements and constraining the format of how quality was perceived locally.

#### Factors influencing the use of NCA data for local QI

Factors which influenced the use of NCA data were cited in 60 (29%) reports. Themes were classified as occurring predominantly at micro, meso or macro levels (Table 2). A recurrent theme was the lack of local capacity or capability for QI. Electronic NCA data collection or analytical tools, such as the NELA and STAG webtools, were popular and reduced local workloads.

**Table 2:** Factors influencing use of data for local QI

**Micro level**	
**Barriers**	**Enablers**
Lack of time and resources	Perceived need to improve from low baseline performance
Lack of QI experience	Embedding data collection into normal practice
Extra data collection needed in addition to NCA data	Multi-faceted approach to data feedback
Lack of awareness of scale of local problems	Leverage of existing networks to disseminate data
Difficulty communicating and collaborating across diverse groups of stakeholders	Use of patients as a ‘technology of persuasion’
Challenges overturning embedded practices	Enthusiasm for QI project
Rotational shift patterns of clinical staff threaten sustainability of projects	
**Meso level**	
**Barriers**	**Enablers**
Challenges collecting data	Supportive digital context
Difficulties accessing existing data	Effective collaboration between managers and clinicians
Difficulties engaging ‘peripheral’ (but important) staff groups like IT or pathology	QI seen as part of normal practice
Lack of incentivisation for clinical staff to perform QI	Sense of community amongst healthcare professionals
Challenges integrating multidisciplinary teams	Avoidance of blame culture
**Macro level**	
**Barriers**	**Enablers**
Challenges regarding data validity/timeliness/completeness	Valid and timely data feedback*
Unconvincing evidence base for improvement	Productive collaborations between hospitals*
Disputed processes of case-mix adjustment	Facilitated sharing of best practice between sites*
Lack of clear actions for improvement provided by NCAs	Central provision of data analytical/visualisation tools*
NCA reports inaccessible to managers/commissioners	Evidence base perceived as strong*
NCA data insufficient for local needs	National performance perceived as weak or variable*
	Financial incentives (e.g. best practice tariffs)
	Relevant and concise reports
	Regulatory/professional pressures to involve patients/public can motivate PROM/PREM collection and use

* denotes factors describing the two NCAs (NHFD and NELA) whose data were most frequently used for local QI

NCAs could support local motivation to engage in QI; the most common use of NCA data was to identify a local issue needing improvement (online Supporting information Table S7). By also demonstrating variation at the national level and/or sharing of best practice, NCAs could illustrate a route to improvement and thereby help overcome local inertia. A credible evidence base and valid data were also crucial to motivate QI; poor results could be explained away if data were not adjusted for local case mix.

The timeliness of NCA feedback was important for QI. A time lag of 1 year to receive NLCA data hindered QI, whereas monthly NHFD feedback provided invaluable positive reinforcement. Effective and accessible data visualisation tools also supported engagement, for example NHFD dashboards provided local sites with time-series displays of their data.

Effective intra-hospital collaboration between groups including managers, information and multidisciplinary clinical teams supported QI but difficult to achieve. Financial incentives, although rarely commented upon in the reports we found, were suggested to help the NHFD best practice tariff was reported to facilitate institutional buy-in and engagement of senior surgeons.

Inter-hospital collaboration was described by 23 (11%) reports. Five formats of collaborative groups were reported: dedicated prospective trials using NCA data (e.g. the Emergency Laparotomy Collaborative); regional QI collaboratives, often comprising trainee doctors (e.g. Liverpool Research Trainee Collaborative); groups leveraging clinical pathways (e.g. regional trauma pathways); Academic Health Science Networks; or between all hospitals participating in a particular NCA. Collaborative activities were popular with local participants, who welcomed social support and peer review of their data or QI ideas but were noted to be potentially difficult to scale if financial support was required.

#### Quality assessment of QI reports

Twenty-five (12%) reports were suitable for quality assessment using the SQUIRE 2.0 guidelines (online Supporting information Table S9). The aims, rationale, available knowledge, intervention(s) and key findings were well described by the majority of these reports. Five (20%) reports specifically addressed unintended consequences of the QI project or adequately described the context where the project took place.

## Discussion

We have conducted the first scoping review of the use of data from UK perioperative NCAs for local QI. We identified 36 separate perioperative NCAs in the UK which had a median duration of 9 years, and we found 209 reports of local QI using data from these NCAs. No QI reports were found for 17 (47%) NCAs, whereas 185 (89%) reports were associated with just six (17%) NCAs. We suggest that this reflects a missed opportunity to support local QI, and that best practice could be spread from the two exemplar NCAs we identified (NHFD and NELA) which measured useful clinical processes, provided valid and timely feedback, facilitated productive collaborative QI efforts using their data involving multiple hospitals and actively sought out and reported case studies of local QI, either through abstract competitions or annual report vignettes.

In keeping with the literature, we found that the nature of NCA feedback influenced how it was used (Ivers et al. 2014). Timely data feedback was reported as enabling local QI, but this strategy was only employed by a minority of NCAs. Feedback delayed by prolonged data validation may be necessary for QA, but is not conducive to rapid improvement cycles. Indeed, only 21 (58%) NCAs explicitly reported taking an approach to support local QI, and all the QI reports we identified only used data from this group of NCAs. This belies the hope that NCA processes designed for assurance could simply be re-purposed for improvement. Funnel plots may reassure hospitals that they lie within two standard deviations from the national mean, prompting little need to improve. Reporting data of individual clinicians may help identify concerning outliers but might not stimulate the team-based activity of QI. We found no NCA supplied sites with targeted local action plans, and passive sharing of data is known to be unlikely to facilitate local QI (Roos-Blom et al. 2019).

Previous literature describes mixed impacts for QI collaboratives (Wells et al. 2018; Hemmila et al. 2018), but we found them to be popular amongst authors of QI reports often because of the crucial social aspects to QI they can support. Inter-hospital collaborative meetings within one QI trial were reported as important for local enthusiasts to share learning and experiences and see that they ‘were not alone’ in seeking to improve care (Stephens et al. 2018). A regional collaborative group noted that meaningful improvement was facilitated by sharing the cultural insights trainee doctors gained by exploring NCA processes in different hospitals (NELA Project Team 2018). However, collaborative projects are expensive in terms of time and resources, and we found not all were successful when results were aggregated at regional/national levels. Future work should explore the optimum models for supporting collaboration within and between hospitals.

It is surprising that only one NCA (NHFD) had a prospective registry of local QI reports, and no NCA reported a QI-output analysis. The abstracts, posters and annual report vignettes which dominated our findings often comprised unstructured narrative, making it hard for others to replicate successful projects. Only a minority of reports described factors influencing their data or projects. Where impacts of QI were explicitly reported, they were almost universally positive, lending weight to suspected publication bias and missing the vital opportunity for sharing learning from negative studies (Ogrinc et al. 2016). These findings echo previous literature describing the lack of routine reporting of QI in comparison with other forms of biomedical research (Bytautas et al. 2017). The nature of how NCAs capture and/or report local QI therefore misses the opportunity to share best practice or as Davidoff argues ‘compromises the ethical obligation to return valuable information to the public’ (Davidoff and Batalden 2005).

Contextual factors beyond the remit of NCAs such as financial constraints, workforce shortages or lack of QI skills may inhibit local QI, but NCA strategies can help overcome these barriers. For example, the data collection burden might be reduced by rationalising datasets to focus more on metrics best suited for local QI; we found that process indicators were more commonly used for QI than outcome metrics. Timely and accessible NCA feedback can facilitate improvement but also needs to be disseminated within hospitals to clinical teams delivering care (Gould et al. 2018); financial incentives can help overcome barriers to collaboration between clinicians and managers. Supporting clinical leads to analyse their data and select improvement strategies may help local teams who are unsure how to deliver change (Sykes et al. 2020).

There are several limitations to this review. By only examining the perioperative setting in the UK, we are unable to comment on measurement systems in other contexts, and it is possible that QI activity might be greater in other specialities or condition-specific audits. We found the majority of QI reports by hand-searching the literature and therefore some reports may have been missed. Further reports may have been published after the searches were made (ending December 2019). Some QI projects may have been reported locally, orally or via social media and would not have been found by our strategy. Furthermore, this review did not look for local improvement per se but for *public reporting* of local QI. We must therefore interpret this review as a minimum estimate of QI activity. However, if more such evidence does exist, it is unlikely to have much reach beyond local contexts. We deliberately excluded national audits which had only released a single report, as although these can and do trigger improvement, they could not have supported the continuous data-driven local QI we were searching for. As discussed above, there is a strong suggestion of publication bias due to under-reporting of QI projects in general and specifically those which did not achieve ‘positive’ results. This bias limits analysis of QI projects’ number, characteristics, impact or learning. Only 12% of QI reports were published in a format appropriate for quality assessment, reflecting the unstructured nature of this literature. Some feedback provided by NCAs to local teams was confidential, hampering our ability to comment on it in this review. Finally, NCAs clearly can and do have impact beyond local continuous QI (for example to support QA or to provide baseline data for research), and local QI can occur without NCA data; the number of QI projects which we found to be publicly reported may not correlate with the wider impact of these NCAs.

## Conclusions

This review provides evidence of missed opportunities for local QI using NCA data in the UK perioperative setting. The two NCAs which were associated with the vast majority of QI reports provided valid and timely feedback, supported collaboration between hospitals and actively sought out local case studies. There was a high likelihood of reporting bias towards projects which achieved a positive impact.

To improve this situation, we would recommend the following strategies. First, NCAs should ensure they measure process metrics amenable to QI. Second, they should deliver feedback in a timely and accessible manner, aimed at teams rather than individuals. Third, feedback should be linked to localised action plans and possibly financial incentives. Fourth, local QI projects and evaluations thereof should be prospectively recorded in accessible registries in order to better share learning from projects achieving both ‘positive’ and ‘negative’ impacts. The interaction between NCA practices with local contexts remains a question for future research, as does the most effective method(s) for promoting collaboration within and between hospitals.

## Supplementary Information

**Additional file 1.**

## Data Availability

Not applicable.

## Abbreviations

HQIP: Healthcare Quality Improvement Partnership
NCA: National Clinical Audit
NHS: National Health Service
PREM: Patient-reported experience measure
PRISMA-ScR: Preferred Reporting Items for Systematic Reviews and Meta-Analyses extension for Scoping Reviews
PROM: Patient-reported outcome measure
PROSPERO: International Prospective Register of Systematic Reviews
QA: Quality assurance
QI: Quality improvement
SHA: Scottish Healthcare Audits
SQUIRE: Standards for Quality Improvement Reporting Excellence
UPCARE: Understanding Practice in Clinical Audit
