# Supplementary material for: A scoping review of local quality improvement using data from UK perioperative National Clinical Audits
Source: Perioper Med (Lond). 2022 Aug 29;11:43. doi: 10.1186/s13741-022-00273-0 (PMC9422140; doi:10.1186/s13741-022-00273-0)
Supplement: Supplementary file 1 — Additional file 1. [file 13741_2022_273_MOESM1_ESM.docx]

# Supporting Information

### Figure S1: Search Strategies

#### MEDLINE

Ovid MEDLINE(R) Daily and Ovid MEDLINE(R) <1946 to Present>

1 (NELA or laparotomy audit or laparotomy collaborative or ELPQuiC or laparotomy pathway or icnarc or "intensive care national audit and research centre" or "case mix programme" or "Bowel Cancer Audit" or NBOCA or NBCA bowel or NJR or national joint registry or NHFD or "national hip fracture database" or NLCA or "National lung cancer audit" or noda audit or National ophthalmology audit or national ophthalmology database or national ophthalmology registry or (nvr and vascular) or national vascular registry or (nnap and neurosurg*) or neurosurgical national audit programme or picanet or paediatric intensive care audit or (NPCA and prostate) or prostate cancer audit or NBSR or national bariatric surgical registry or HANA audit or "head and neck cancer audit" or DAHNO or "fractured neck of femur audit" or nchda or national congenital heart disease audit or nacsa or cardiac surgery registry or cardiac surgery audit or ((BAUS or British association of urological surgeons) and (audit or registry or database or observatory)) or national proms programme or patient reported outcome measures programme or (tarn and audit) or major trauma audit or "National Comparative Audit of Blood Transfusion" or Scottish Hip Fracture Audit or Scottish Arthroplasty Project or Audit of Critical Care in Scotland or Audit of Trauma Management in Scotland).mp. [mp=title, abstract, original title, name of substance word, subject heading word, keyword heading word, protocol supplementary concept word, rare disease supplementary concept word, unique identifier, synonyms] (743)

2 exp Blood Transfusion/ (97982)

3 exp Trauma Centers/ or exp Multiple Trauma/ (21680)

4 exp Prostatectomy/ or exp Cystectomy/ or exp Nephrectomy/ or exp Urinary Incontinence, Stress/ (85418)

5 exp Thoracic surgery/ (12963)

6 exp Laparotomy/ (19667)

7 intensive care.mp. or exp Critical Care/ (184560)

8 exp Rectal Neoplasms/ or exp Colonic Neoplasms/ or exp Colorectal Neoplasms/ or exp Intestinal Neoplasms/ (224221)

9 joint replacement.mp. or exp Arthroplasty, Replacement/ (52802)

10 exp Hip Fractures/ (23376)

11 lung cancer.mp. or exp Lung Neoplasms/ (264089)

12 ophthalmology.mp. or exp Ophthalmology/ (37137)

13 exp Vascular Surgical Procedures/ or vascular surgery.mp. (250536)

14 neurosurgery.mp. or exp Neurosurgery/ (29356)

15 prostate cancer.mp. or exp Prostatic Neoplasms/ (151444)

16 bariatric surgery.mp. or exp Bariatric Surgery/ (29457)

17 (head and neck cancer).mp. [mp=title, abstract, original title, name of substance word, subject heading word, keyword heading word, protocol supplementary concept word, rare disease supplementary concept word, unique identifier, synonyms] (22268)

18 exp Femoral Fractures/ (38324)

19 exp Heart Defects, Congenital/ or congenital heart disease.mp. (160991)

20 patient reported outcome measures.mp. or exp Patient Reported Outcome Measures/ (3404)

21 medical audit.mp. or exp Medical Audit/ (17818)

22 registry.mp. or exp Registries/ (138447)

23 exp Database Management Systems/ (8033)

24 21 or 22 or 23 (163601)

25 exp United Kingdom/ or UK.mp. or Great Britain.mp. or British.mp. or England.mp. or English.mp. or Scotland.mp. or Scottish.mp. or Ireland.mp. or Irish.mp. or Wales.mp. or welsh.mp. [mp=title, abstract, original title, name of substance word, subject heading word, keyword heading word, protocol supplementary concept word, rare disease supplementary concept word, unique identifier, synonyms] (2374421)

26 exp Quality Improvement/ or exp Total Quality Management/ or (pdsa or pdca or lean or six sigma or QI or total quality management or tqm or continuous quality management or CQM or continuous quality improvement or CQI or (quality adj3 improv*)).mp. [mp=title, abstract, original title, name of substance word, subject heading word, keyword heading word, protocol supplementary concept word, rare disease supplementary concept word, unique identifier, synonyms] (192671)

27 2 or 3 or 4 or 5 or 6 or 7 or 8 or 9 or 10 or 11 or 12 or 13 or 14 or 15 or 16 or 17 or 18 or 19 or 20 (1597924)

28 24 and 25 and 27 (3440)

29 1 or 28 (3980)

30 26 and 29 (175)

***************************

#### EMBASE

Database: Embase <1980 to 2017 Week 52>

Search Strategy:

--------------------------------------------------------------------------------

1 (NELA or laparotomy audit or laparotomy collaborative or ELPQuiC or laparotomy pathway or icnarc or "intensive care national audit and research centre" or "case mix programme" or "Bowel Cancer Audit" or NBOCA or NBCA bowel or NJR or national joint registry or NHFD or "national hip fracture database" or NLCA or "National lung cancer audit" or noda audit or National ophthalmology audit or national ophthalmology database or national ophthalmology registry or (nvr and vascular) or national vascular registry or (nnap and neurosurg*) or neurosurgical national audit programme or picanet or paediatric intensive care audit or (NPCA and prostate) or prostate cancer audit or NBSR or national bariatric surgical registry or HANA audit or "head and neck cancer audit" or DAHNO or "fractured neck of femur audit" or nchda or national congenital heart disease audit or nacsa or cardiac surgery registry or cardiac surgery audit or ((BAUS or British association of urological surgeons) and (audit or registry or database or observatory)) or national proms programme or patient reported outcome measures programme or (tarn and audit) or major trauma audit or "National Comparative Audit of Blood Transfusion" or Scottish Hip Fracture Audit or Scottish Arthroplasty Project or Audit of Critical Care in Scotland or Audit of Trauma Management in Scotland).mp. [mp=title, abstract, heading word, drug trade name, original title, device manufacturer, drug manufacturer, device trade name, keyword, floating subheading word] (1638)

2 exp Blood Transfusion/ (158019)

3 exp Trauma Centers/ or exp Multiple Trauma/ (96953)

4 exp Prostatectomy/ or exp Cystectomy/ or exp Nephrectomy/ or exp Urinary Incontinence, Stress/ (145464)

5 exp Thoracic surgery/ (512374)

6 exp Laparotomy/ (68104)

7 intensive care.mp. or exp Critical Care/ (706601)

8 exp Rectal Neoplasms/ or exp Colonic Neoplasms/ or exp Colorectal Neoplasms/ or exp Intestinal Neoplasms/ (341888)

9 joint replacement.mp. or exp Arthroplasty, Replacement/ (16507)

10 exp Hip Fractures/ (30881)

11 lung cancer.mp. or exp Lung Neoplasms/ (348275)

12 ophthalmology.mp. or exp Ophthalmology/ (55210)

13 exp Vascular Surgical Procedures/ or vascular surgery.mp. (404565)

14 neurosurgery.mp. or exp Neurosurgery/ (234729)

15 prostate cancer.mp. or exp Prostatic Neoplasms/ (218422)

16 bariatric surgery.mp. or exp Bariatric Surgery/ (34726)

17 (head and neck cancer).mp. [mp=title, abstract, heading word, drug trade name, original title, device manufacturer, drug manufacturer, device trade name, keyword, floating subheading word] (48760)

18 exp Femoral Fractures/ (24861)

19 exp Heart Defects, Congenital/ or congenital heart disease.mp. (144310)

20 patient reported outcome measures.mp. or exp Patient Reported Outcome Measures/ (8185)

21 medical audit.mp. or exp Medical Audit/ (48277)

22 registry.mp. or exp Registries/ (192605)

23 exp Database Management Systems/ (209)

24 21 or 22 or 23 (239506)

25 exp United Kingdom/ or UK.mp. or Great Britain.mp. or British.mp. or England.mp. or English.mp. or Scotland.mp. or Scottish.mp. or Ireland.mp. or Irish.mp. or Wales.mp. or welsh.mp. [mp=title, abstract, heading word, drug trade name, original title, device manufacturer, drug manufacturer, device trade name, keyword, floating subheading word] (892076)

26 exp Quality Improvement/ or exp Total Quality Management/ or (pdsa or pdca or lean or six sigma or QI or total quality management or tqm or continuous quality management or CQM or continuous quality improvement or CQI or (quality adj3 improv*)).mp. [mp=title, abstract, heading word, drug trade name, original title, device manufacturer, drug manufacturer, device trade name, keyword, floating subheading word] (251274)

27 2 or 3 or 4 or 5 or 6 or 7 or 8 or 9 or 10 or 11 or 12 or 13 or 14 or 15 or 16 or 17 or 18 or 19 or 20 (3060420)

28 24 and 25 and 27 (7023)

29 1 or 28 (8119)

30 26 and 29 (398)

***************************

#### HMIC

Database: HMIC Health Management Information Consortium <1979 to September 2017>

Search Strategy:

--------------------------------------------------------------------------------

1 (NELA or laparotomy audit or laparotomy collaborative or ELPQuiC or laparotomy pathway or icnarc or "intensive care national audit and research centre" or "case mix programme" or "Bowel Cancer Audit" or NBOCA or NBCA bowel or NJR or national joint registry or NHFD or "national hip fracture database" or NLCA or "National lung cancer audit" or noda audit or National ophthalmology audit or national ophthalmology database or national ophthalmology registry or (nvr and vascular) or national vascular registry or (nnap and neurosurg*) or neurosurgical national audit programme or picanet or paediatric intensive care audit or (NPCA and prostate) or prostate cancer audit or NBSR or national bariatric surgical registry or HANA audit or "head and neck cancer audit" or DAHNO or "fractured neck of femur audit" or nchda or national congenital heart disease audit or nacsa or cardiac surgery registry or cardiac surgery audit or ((BAUS or British association of urological surgeons) and (audit or registry or database or observatory)) or national proms programme or patient reported outcome measures programme or (tarn and audit) or major trauma audit or "National Comparative Audit of Blood Transfusion" or Scottish Hip Fracture Audit or Scottish Arthroplasty Project or Audit of Critical Care in Scotland or Audit of Trauma Management in Scotland).mp. [mp=title, other title, abstract, heading words] (79)

2 medical audit.mp. or exp Medical Audit/ (2370)

3 registry.mp. or exp Registries/ (1045)

4 exp Database Management Systems/ (28)

5 2 or 3 or 4 (3431)

6 exp United Kingdom/ or UK.mp. or Great Britain.mp. or British.mp. or England.mp. or English.mp. or Scotland.mp. or Scottish.mp. or Ireland.mp. or Irish.mp. or Wales.mp. or welsh.mp. [mp=title, other title, abstract, heading words] (82856)

7 exp Quality Improvement/ or exp Total Quality Management/ or (pdsa or pdca or lean or six sigma or QI or total quality management or tqm or continuous quality management or CQM or continuous quality improvement or CQI or (quality adj3 improv*)).mp. [mp=title, other title, abstract, heading words] (10443)

8 wounds & injuries/ or back injuries/ or head injuries/ or heat injuries/ or personal injury/ or work related injuries/ or accident & emergency patients/ or accidents/ or debridement/ or trauma care/ or trauma centres/ (1695)

9 blood transfusion/ (281)

10 prostate cancer/ or prostatectomy/ (465)

11 cancer/ or alimentary tract cancer/ or bladder cancer/ or brain cancer/ or bronchial cancer/ or gastrointestinal cancer/ or intestinal cancer/ or laryngeal cancer/ or lung cancer/ or mesothelioma/ or oesophageal cancer/ or prostate cancer/ or renal cancer/ or thyroid cancer/ (7285)

12 exp Thoracic surgery/ (18)

13 laparotomy/ or abdominal surgery/ (55)

14 thoracic surgery/ or thoracic surgery units/ (23)

15 critical care/ or critical care units/ or high dependency care/ or high dependency units/ or intensive care/ or intensive care units/ (1485)

16 hip surgery/ or hip joints/ or hip units/ (249)

17 arthroplasty/ or joint replacement surgery/ (94)

18 knee joint replacement/ (65)

19 exp Hip surgery/ or exp Hip joints/ (249)

20 exp Ophthalmology units/ or exp Ophthalmology/ or exp Ophthalmology services/ (732)

21 vascular surgery/ (43)

22 neurosurgery/ or brain surgery/ or neurosurgeons/ or neurosurgical health services/ or neurosurgical hospitals/ or neurosurgical units/ (75)

23 exp Bariatric surgery/ (71)

24 (head and neck cancer).mp. [mp=title, other title, abstract, heading words] (56)

25 femoral fracture.mp. (7)

26 congenital heart disease.mp. or exp Congenital heart abnormalities/ (92)

27 patient reported outcome measures.mp. (89)

28 8 or 9 or 10 or 11 or 12 or 13 or 14 or 15 or 16 or 17 or 18 or 19 or 20 or 21 or 22 or 23 or 24 or 25 or 26 or 27 (12199)

29 5 and 6 and 28 (111)

30 1 or 29 (186)

31 7 and 30 (7)

***************************

#### WEB OF SCIENCE


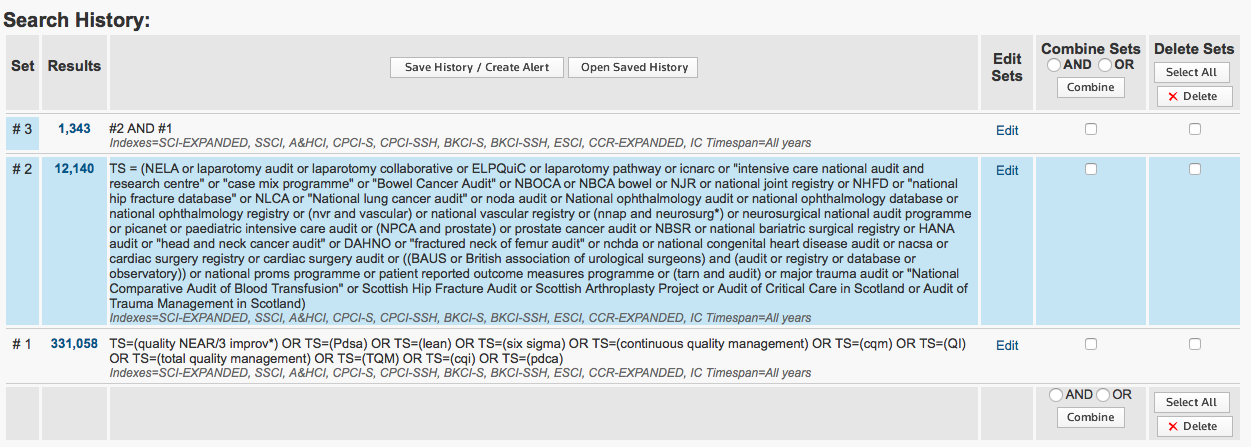


#### GOOGLE SCHOLAR

“national emergency laparotomy audit” OR “”NELA” OR "intensive care national audit and research centre" OR “ICNARC” OR “CMP” OR "national bowel cancer audit" OR “NBOCA” OR “national joint registry” OR “NJR” OR "national hip fracture database" OR “NHFD” OR "National lung cancer audit" OR “NLCA” OR “National ophthalmology audit” “NOA” OR “national ophthalmology database” OR “national vascular registry” “NVR” OR “VSQIP” OR “neurosurgical national audit programme” OR “NNAP” OR “paediatric intensive care audit” OR “PICANET” OR “national prostate cancer audit” OR “NPCA” OR “national bariatric surgical registry” OR “NBSR” OR "head and neck cancer audit" “HANA” OR “DAHNO” OR "fractured neck of femur audit" “FNF” OR “national congenital heart disease audit” OR “national adult cardiac surgery audit” OR “British association of urological surgeons audit” OR “BAUS” OR “patient reported outcome measures programme” OR “PROMS” OR “major trauma audit “ OR “TARN” OR "National Comparative Audit of Blood Transfusion" OR “NBT” OR “Scottish Hip Fracture Audit” OR “SHFA” OR “Scottish Arthroplasty Project” OR “SAP” OR “Audit of Critical Care in Scotland” OR “SICSAG” OR “Audit of Trauma Management in Scotland” OR “STAG” AND “quality improvement” OR “pdsa” OR pdca” OR “six sigma” OR “quality management”

### Figure S2: Data Charting Template

| First author surname | |
| --- | --- |
| Year of publication | |
| Country | |
| Specific location | |
| Manuscript type | 1. Conference abstract / poster 2. QI report 3. Empirical evaluation of QI intervention(s) 4. Commentary / editorial / review 5. Systematic review 6. Letter 7. Webpage 8. Other |
| Audience targeted (e.g. predominant journal readership) | 1. Anaesthesia 2. Surgery 3. Physicians 4. Nursing 5. Other clinical – please specify 6. QI / HSR 7. Management / commissioners |
| National clinical audit (s) used | |
| NCA indicator(s) used | 1. Structure 2. Process 3. Outcome |
| Quality domain(s) attempted to be improved | 1. Safety 2. Effectiveness 3. Patient experience 4. Efficiency / productivity 5. Other |
| Scale of improvement | 1. Single hospital / trust 2. Collaboration (i.e. more than one trust) 3. Other |
| Improvement team  (tick all that apply) | 1. Anaesthesia (trainees) 2. Anaesthesia (consultants) 3. Surgery (trainees) 4. Surgery (consultants) 5. Nursing staff 6. AHPs 7. Non-clinical managers 8. PPI |
| Purpose of using NCA data  (tick all that apply) | 1. Identify target for QI 2. Prioritise between QI projects 3. Initiate a QI project 4. Monitor/evaluate progress of a QI project already underway 5. Other |
| Activities involving NCA data  (tick all that apply) | 1. Collection of additional data 2. Local analysis of NCA data 3. Local dissemination of NCA data 4. Other |
| QI intervention  (tick all that apply) | 1. New / altered Pathway 2. New / altered Care bundle 3. New / altered Policy / Guideline 4. Other |
| Evaluation of QI project  (tick all that apply) | 1. Quantitative 2. Qualitative 3. Costs 4. Other |
| QI project deemed successful | 1. Yes 2. No |
| Influencing factors – internal to organisation | 1. Barriers 2. Enablers |
| Influencing factors – external to organisation | 1. Barriers 2. Enablers |
| Benefits of QI project |  |
| Harms of QI project |  |
| Evidence of spread / scaling |  |
| Other |  |

### Figure S3: Identification of Perioperative NCAs

### ***
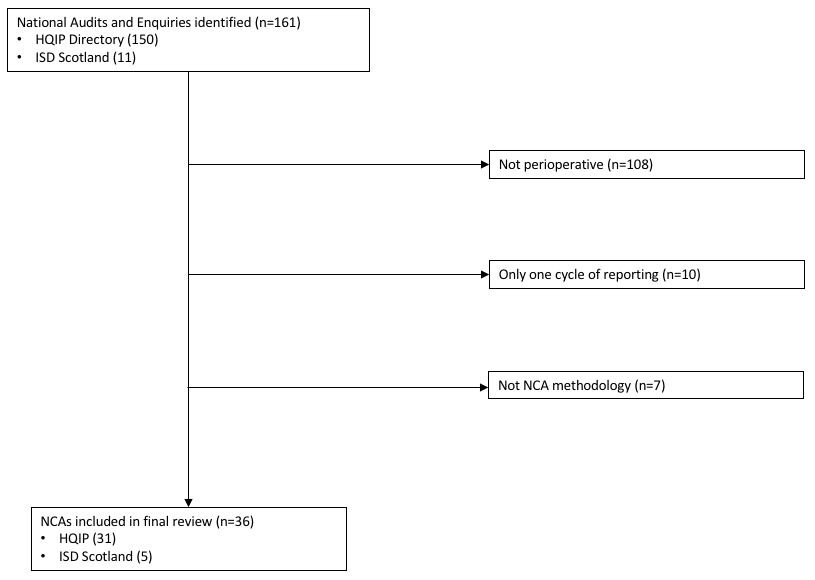
***

### Figure S4: QI Activity by Duration of NCA

### Table S1: Perioperative NCAs identified during Phase One

| **Acronym** | **Full name** |
| --- | --- |
| ACS | Adult Cardiac Surgery |
| BAUS-FSUI | British Association of Urological Surgery (BAUS) Audits - Female Stress Urinary Incontinence Audit |
| BAUS-RPA | British Association of Urological Surgery (BAUS) Audits - Radical Prostatectomy Audit |
| BAUS-Cyst | British Association of Urological Surgery (BAUS) Audits - Cystectomy |
| BAUS-Neph | British Association of Urological Surgery (BAUS) Audits - Nephrectomy audit |
| BAUS-PCNL | British Association of Urological Surgery (BAUS) Audits - Percutaneous Nephrolithotomy |
| BAUS-Ureth | British Association of Urological Surgery (BAUS) Audits -Urethroplasty Audit |
| BCIR | Breast and Cosmetic Implant Registry |
| ICNARC-CMP | Case Mix Programme |
| PROMs | Elective Surgery (National Pateint Reported Outcome Measures (PROMs) Programme) |
| BAETS | Endocrine and Thyroid National Audit |
| NHFD | Falls and Fragility Fractures Audit programme |
| FNF | Fractured Neck of Femur (care in emergency departments) |
| CRANE | Cleft Registry and Audit Network |
| DAHNO | Head and Neck Cancer Audit |
| TARN | Major Trauma Audit |
| NABCOP | National Audit of Breast Cancer in Older People |
| NBSR | National Bariatric Surgery Registry |
| NBOCA | National Bowel Cancer |
| NBT | National Comparative Audit of Blood Transfusion programme (elective surgery) |
| CHD | National Congenital Heart Disease |
| NELA | National Emergency Laparotomy Audit |
| NJR | National Joint Registry |
| NLCA | National Lung Cancer Audit |
| NOGCA | National Oesophago-Gastric Cancer Audit |
| NOA | National Ophthalmology Audit |
| NPCA | National Prostate Cancer Audit |
| NVR | National Vascular Registry |
| NNAP | Neurosurgical National Audit Programme |
| PICANet | Paediatric Intensive Care Audit Network |
| PQIP | Perioperative Quality Improvement Programme |
| SAP | Scottish Arthroplasty project |
| SICSAG | Scottish Intensive Care Society Audit Group |
| STAG | Scottish Trauma Audit Group |
| SHFA | Scottish Hip Fracture Audit |
| SAIVM | Scottish Audit of Intracranial Vascular Malformations |

### Table S2: Summary Features of NCAs

|  |  | **Number (denominator*)** | **%** |
| --- | --- | --- | --- |
| **Structures** | **NCAs in England & Wales** | 31 (36) | 86 |
|  | **NCAs in Scotland** | 5 (36) | 14 |
|  | **Providers in England & Wales:**  NHS Digital  NHS Blood & Transplant  HQIP  Surgical Royal Colleges / Specialties  Perioperative Professional Bodies | 4 (31)  1 (31)  1 (31)  15 (31)  10 (31) | 13  3  3  48  32 |
|  | **Self-assessments available** | 21 (36) | 58 |
|  | **Self-assessed as adopting a ‘QI approach’** | 18 (21) | 86 |
|  | **Focused on a specific procedure** | 12 (36) | 33 |
|  | **Focused on a specific condition** | 13 (36) | 36 |
|  | **Focused on a specific specialty** | 6 (36) | 17 |
|  | **Focused on perioperative issues** | 5 (36) | 14 |
|  | **Collects data on Structures** | 11 (36) | 31 |
|  | **Collects data on Processes** | 36 (36) | 100 |
|  | **Collects data on Outcomes** | 36 (36) | 100 |
|  | **Measures at the level of hospital** | 36 (36) | 100 |
|  | **Measures at the level of individuals** | 16 (36) | 44 |
| **Processes** | **Provision of targeted action plans for hospitals** | 0 (21) | 0 |
|  | **Provision of results in real-time** | 8 (21) | 38 |
|  | **Provision of a care bundle** | 0 (21) | 0 |
|  | **Introduction of PROMs** | 5 (36) | 14 |
|  | **Best Practice Tariff** | 4 (36) | 11 |
|  | **Tools (e.g. clinical decision aids)** | 1 (21) | 5 |
|  | **Education** | 16 (36) | 44 |
|  | **Positive deviance** | 9 (31) | 29 |
|  | **Abstract competitions** | 5 (36) | 14 |
| **Outcomes**  **(national)** | **Improved clinical processes / outcomes over NCA lifetime** | 35/36 | 97 |
|  | **Contributed data to NHS quality dashboard** | 9 (16) | 56 |
|  | **Contributed to CQC** | 6/16 | 38 |
|  | **Contributed to NICE guideline** | 3/16 | 19 |
| **Outcomes**  **(local)** | **Data used for local QI** | 16/21 | 76 |
|  | **Example local QI projects available in report/website** | 8/36 | 22 |

*Self-assessments were only available for 21 NCAs but some data were available elsewhere, hence the denominators vary across fields.

### Table S3: Features of Individual NCAs

| **Code** | **Type of NCA** | **Provider** | **Start date** | **NHSe quality accounts** | **Best Practice Tariff** | **Self-assessment available** | **Reporting at level of individual clinician** | **QI approach stated in protocol or website** | **Education sessions** | **Examples of local QI cited on website / report** | **Data used for local QI? (self-assessment)** | **Real-time feedback** |
| --- | --- | --- | --- | --- | --- | --- | --- | --- | --- | --- | --- | --- |
| **ACS** | Specialty | National Institute for Cardiovascular Outcomes Research (NICOR) | 1997 | Yes | No | Yes | Yes | Yes | Yes | No | Yes | No |
| **BAUS-FSUI** | Procedure | British Association of Urological Surgeons | 2015 | Yes | No | No | Yes | No | No | No | N/A | N/A |
| **BAUS-RPA** | Procedure | British Association of Urological Surgeons | 2015 | Yes | No | No | Yes | No | No | No | N/A | N/A |
| **BAUS-Cyst** | Procedure | British Association of Urological Surgeons | 2016 | Yes | No | No | Yes | No | No | No | N/A | N/A |
| **BAUS-Neph** | Procedure | British Association of Urological Surgeons | 2013 | Yes | No | No | Yes | No | No | No | N/A | N/A |
| **BAUS-PCNL** | Procedure | British Association of Urological Surgeons | 2013 | Yes | No | No | Yes | No | Yes | No | N/A | N/A |
| **BAUS-Ureth** | Procedure | British Association of Urological Surgeons | 2015 | Yes | No | No | Yes | No | No | No | N/A | N/A |
| **ICNARC-CMP** | Perioperative | Intensive Care National Audit and Research Centre - Case Mix Programme | 1994 | Yes | No | Yes | No | Yes | Yes | Yes | Yes | No |
| **PROMs** | Procedure | NHS Digital | 2009 | Yes | No | Yes | No | No | Yes | Yes | Yes | No |
| **BAETS** | Specialty | British Association of Endocrine and Thyroid Surgeons | 2012 | Yes | No | No | Yes | No | No | No | N/A | N/A |
| **NHFD** | Condition | Royal College of Physicians of London | 2007 | Yes | Yes | Yes | No | No | No | Yes | Yes | Yes |
| **FNF** | Condition | Royal College of Emergency Medicine | 2003 | Yes | No | No | No | No | No | No | N/A | N/A |
| **DAHNO** | Specialty | Saving Faces - The Facial Surgery Research Foundation | 2003 | Yes | No | Yes | Yes | Yes | Yes | No | Yes | No |
| **TARN** | Condition | The Trauma Audit and Research Network (TARN) | 2001 | Yes | Yes | Yes | No | Yes | Yes | No | Yes | No |
| **NBSR** | Specialty | British Obesity and Metabolic Surgery Society (BOMSS) | 2008 | Yes | No | No | Yes | No | No | No | N/A | N/A |
| **NBOCA** | Condition | NHS Digital | 2003 | Yes | No | Yes | No | Yes | Yes | No | Yes | Yes |
| **NBT** | Perioperative | NHS Blood and Transplant | 2015 | Yes | No | Yes | No | No | No | No | No | No |
| **CHD** | Condition | National Institute for Cardiovascular Outcomes Research (NICOR) | 2000 | Yes | No | Yes | No | Yes | No | No | No | No |
| **NELA** | Procedure | Royal College of Anaesthetists | 2012 | Yes | Yes | Yes | No | Yes | Yes | Yes | Yes | Yes |
| **NJR** | Procedure | Healthcare Quality Improvement Partnership (HQIP) | 2002 | Yes | Yes | Yes | Yes | Yes | No | No | Yes | No |
| **NLCA** | Condition | Royal College of Physicians | 2005 | Yes | No | Yes | No | Yes | Yes | Yes | Yes | Yes |
| **NOGCA** | Condition | NHS Digital | 2011 | Yes | No | Yes | Yes | Yes | Yes | No | Yes | No |
| **NOA** | Procedure | Royal College of Ophthalmologists | 2014 | Yes | No | No | Yes | No | No | No | N/A | No |
| **NPCA** | Condition | Royal College of Surgeons of England (Clinical Effectiveness Unit) | 2014 | Yes | No | Yes | No | Yes | No | No | No | No |
| **NVR** | Specialty | Royal College of Surgeons of England | 2012 | Yes | No | Yes | Yes | Yes | Yes | No | Yes | No |
| **NNAP** | Specialty | Society of British Neurological Surgeons | 2013 | Yes | No | No | Yes | Yes | No | No | N/A | N/A |
| **PICANet** | Perioperative | University of Leeds | 2004 | Yes | No | Yes | No | No | Yes | No | Yes | No |
| **SAP** | Procedure | Public Health Scotland | 2001 | N/A | N/A | Yes | Yes | Yes | Yes | No | No | Yes |
| **SICSAG** | Perioperative | NHS National Services Scotalnd | 1995 | N/A | N/A | Yes | No | Yes | Yes | No | Yes | Yes |
| **STAG** | Condition | NHS National Services Scotalnd | 2011 | N/A | N/A | Yes | No | Yes | Yes | Yes | Yes | Yes |
| **SHFA** | Condition | s | 2012 | N/A | N/A | Yes |  | Yes | Yes | Yes | Yes | Yes |
| **SAIVM** | Condition | NHS National Services Scotalnd | 1999 | N/A | N/A | Yes | No | Yes | No | No | No | No |
| **BCIR** | Procedure | NHS Digital | 2012 | No | No | No | No | No | No | No | N/A | No |
| **CRANE** | Condition | Clinical Effectiveness Unit, The Royal College of Surgeons of England | 2012 | No | No | No | No | No | No | No | N/A | No |
| **NABCOP** | Condition | Clinical Effectiveness Unit - Royal College of Surgeons | 2017 | Yes | No | No | No | No | No | No | N/A | No |
| **PQIP** | Perioperative | Royal College of Anaesthetists | 2017 | Yes | No | No | No | Yes | Yes | Yes | N/A | N/A |

### Table S4: Summary of Included QI Reports

| **First Author** | **Year** | **NCA** | **Report type** | **Study description** | **Location** | **No. of sites** | **Impact** |
| --- | --- | --- | --- | --- | --- | --- | --- |
| Aggarwal^1^ | 2017 | NELA | Commentary / editorial | Commentary of the Emergency Laparotomy Collaborative (ELC) | Wessex, Kent, Surrey, Sussex | Single | Not stated |
| Gousia^2^ | 2017 | NELA | Evaluation of QI project | Interim evaluation of the ELC | Wessex, Kent, Surrey, Sussex | Multiple | Positive impact |
| Aveling^3^ | 2017 | NLCA | Commentary / editorial | Comparative case studies of clinical community model | ILCOP | Multiple | Mixed impacts |
| Russell^4^ | 2014 | NLCA | Evaluation of QI project | Multicentre RCT of Improving Lung Cancer Outcomes Project (ILCOP) | ILCOP | Multiple | Positive impact |
| Mayor^5^ | 2012 | NLCA | Commentary / editorial | Commentary of ILCOP | ILCOP | Multiple | Positive impact |
| RCP^6^ | 2012 | NLCA | Unstructured report | Case study of ILCOP | Burton | Single | Positive impact |
| RCP^6^ | 2012 | NLCA | Unstructured report | Case study of ILCOP | Salford | Single | Positive impact |
| RCP^6^ | 2012 | NLCA | Unstructured report | Case study of ILCOP | Royal Cornwall | Single | Not stated |
| RCP^6^ | 2012 | NLCA | Unstructured report | Case study of ILCOP | King's Lynn | Single | Not stated |
| Armstrong^7^ | 2012 | NLCA | Evaluation of QI project | Evaluation of ILCOP | ILCOP | Multiple | Positive impact |
| Aveling^8^ | 2012 | NLCA | Evaluation of QI project | Ethnographic case study of ILCOP | ILCOP | Multiple | Not stated |
| Armstrong^9^ | 2012 | NLCA, NVR | Secondary analysis of empirical data | Optimising patient involvement in QI - ethnographic study | ILCOP and AAQIP | Multiple | Positive impact |
| Pearse^10^ | 2014 | NELA | Protocol | Protocol for EPOCH trial: multicentre step-wedged RCT of QI intervention using NELA data | EPOCH | Multiple | Not stated |
| Basser^11^ | 2015 | PROMs | Unstructured report | Establishing Enhanced Recovery | Barnsley | Single | Positive impact |
| Basser^11^ | 2015 | PROMs | Unstructured report | Establishing Enhanced Recovery | Bath | Single | Positive impact |
| Basser^11^ | 2015 | PROMs | Unstructured report | Improving post-op pain | Derby | Single | Positive impact |
| Basser^11^ | 2015 | PROMs | Unstructured report | Disseminating linked data | East Kent | Single | Not stated |
| Basser^11^ | 2015 | PROMs | Unstructured report | Investigating poor performance | Harrogate | Single | Not stated |
| Basser^11^ | 2015 | PROMs | Unstructured report | Changing surgical techniques | Northumbria | Single | Positive impact |
| Basser^11^ | 2015 | PROMs | Unstructured report | Analysing poor performance | RNOH | Single | Not stated |
| Basser^11^ | 2015 | PROMs | Unstructured report | Shared Decision Making | York | Multiple | Not stated |
| Basser^11^ | 2015 | PROMs | Unstructured report | Regional Quality Observatory | NEQOS | Multiple | Not stated |
| Partridge^12^ | 2016 | PROMs, NJR | QI report | Using PROMS to guide change in surgical technique | Northumbria | Single | Positive impact |
| Graham^13^ | 2017 | NHFD | QI report | Reducing return to theatre for hip fracture patients | Bath | Single | Positive impact |
| Lisk^14^ | 2017 | NHFD | QI report | Reducing mortality from hip fractures | Ashford | Single | Positive impact |
| Chamberlain^15^ | 2015 | NHFD | QI report | New pathway to improve hip fracture care | UCLH | Single | Positive impact |
| Gupta^16^ | 2014 | NHFD | QI report | New acute hip unit led by geriatricians | Carmarthen | Single | Positive impact |
| NHFD team^17^ | 2016 | NHFD | Unstructured report | Case study of pathway redesign to improve mortality | Worthing | Single | Positive impact |
| NHFD team^17^ | 2016 | NHFD | Unstructured report | Case study of improved MDT working | North Durham | Single | Not stated |
| NHFD team^17^ | 2016 | NHFD | Unstructured report | Case study of pathway redesign | Royal Berkshire | Single | Positive impact |
| NHFD team^17^ | 2016 | NHFD | Unstructured report | Case study of improving analgesia and anaesthesia | Basildon | Single | Positive impact |
| Hawkes^18^ | 2015 | NHFD | QI report | Increasing trauma lists to improve time to surgery | Bolton | Single | Positive impact |
| Middleton^19^ | 2016 | NHFD | QI report | Improving hip fracture outcomes with integrated orthogeriatric care | Epsom | Single | Not stated |
| Howell^20^ | 2017 | NVR | Commentary / editorial | Commentary of national QI programme (AAAQIP) | AAA-QIP | Multiple | Positive impact |
| Taylor^21^ | 2016 | DAHNO, NBOCA, NLCA, NOGCA | Commentary / editorial | Mixed methods study of how feedback from English NCAs is used | England | Multiple | Positive impact |
| Davenport^22^ | 2010 | TARN | Evaluation of QI project | Evaluation of performance improvement programme for major trauma | London | Single | Positive impact |
| STAG report^23^ | 2017 | STAG | Unstructured report | Case study of using STAG data | STAG | Single | Not stated |
| STAG report^23^ | 2017 | STAG | Unstructured report | Case study of using STAG data | STAG | Single | Not stated |
| STAG report^23^ | 2017 | STAG | Unstructured report | Case study of using STAG data | STAG | Single | Not stated |
| STAG report^23^ | 2017 | STAG | Unstructured report | Case study of using STAG data | STAG | Single | Not stated |
| STAG report^23^ | 2017 | STAG | Unstructured report | Case study of using STAG data | Hairmyres | Single | Not stated |
| STAG report^23^ | 2017 | STAG | Unstructured report | Case study of using STAG data | Lothian | Single | Not stated |
| National Services Scotland^24^ | 2017 | SHFA | Unstructured report | Case study of orthogeriatric nurse practitioner improving hip fracture care | Forth valley | Single | Not stated |
| National Services Scotland^25^ | 2017 | SHFA | Unstructured report | Case study of clinical services manager improving services | Lothian | Single | Not stated |
| National Services Scotland^25^ | 2017 | SHFA | Unstructured report | Case study of physiotherapy manager improving services | Wishaw | Single | Not stated |
| National Services Scotland^26^ | 2017 | SHFA | Unstructured report | Improving nerve block provision for hip fracture | Glasgow | Single | Not stated |
| Jimenez^27^ | 2011 | NLCA | Abstract | Conference report of ILCOP | ILCOP | Multiple | Not stated |
| Patel^28^ | 2017 | NELA | Poster | Local experience of EPOCH | Morecambe Bay | Single | Positive impact |
| Shilston^29^ | 2016 | NELA | Abstract | Using care bundles from EPOCH | Stoke Mandeville | Single | Not stated |
| Kersan^30^ | 2016 | NELA | Abstract | Benchmakring Scottish performance using NELA critieria | Monklands | Single | Not stated |
| Mcdermott^31^ | 2016 | NELA | Poster | Improving recording of pre-op risk assessment | Exeter | Single | No impact |
| Body^32^ | 2016 | NELA | Abstract | Implementation of pathway for high-risk laparotomy patients | King's College | Single | Not stated |
| Cartwright^33^ | 2015 | NELA | Abstract | Improving pre-op risk assessment | Northwick Park | Single | Not stated |
| Baloch^34^ | 2015 | NELA | Abstract | Designing and implementing a new pathway | Warrington | Single | Not stated |
| Coombs^32^ | 2017 | NELA | Abstract | Protective lung ventilation for laparotomy patients | Royal Cornwall | Single | No impact |
| Garland^32^ | 2017 | NELA | Abstract | Impementing a new pathway for laparotomy patients | King's College London | Single | Positive impact |
| O'carroll^32^ | 2017 | NELA | Abstract | Cumulative marginal gains to improve care for emergency laparotomy patients | Maidstone | Single | Not stated |
| Tapley^32^ | 2017 | NELA | Abstract | Peri-operative risk calculation and reduction in NELA patients | Portsmouth | Single | Positive impact |
| Walker^32^ | 2017 | NELA | Abstract | Using NELA data to produce sustained changes | Homerton | Single | Positive impact |
| Bailey^35^ | 2016 | NELA | Abstract | Using NELA data to create a new culture in peri-operative care | Homerton | Single | Positive impact |
| Chong^35^ | 2016 | NELA | Abstract | Implementing NELA recommendations in a District General Hospital | Kingston | Single | Positive impact |
| Dixon^35^ | 2016 | NELA | Abstract | Postoperative pneumonia prevention in NELA patients | Gateshead | Single | Positive impact |
| Hayward^35^ | 2016 | NELA | Abstract | Improving risk assessment for NELA patients | Southampton | Single | Positive impact |
| Madden^35^ | 2016 | NELA | Abstract | Management of sepsis in NELA patients | Coventry | Single | Not stated |
| Pachter^35^ | 2016 | NELA | Abstract | Improving care using NELA recommendations | Sunderland | Single | Not stated |
| Williams^35^ | 2016 | NELA | Abstract | Administering NELA in the context of a previously successful QI care bundle: the loss of marginal gains | Dunn | Single | Negative impact |
| Singh^36^ | 2016 | NELA | Abstract | QI through EPOCH to recude emergency imaging delays | Furness | Single | Positive impact |
| Makvana^36^ | 2016 | NELA | Abstract | QI thorugh EPOCH to improve compliance with sepsis guidelines | Furness | Single | No impact |
| Mcilveen^36^ | 2016 | NELA | Abstract | Comparison of operative and non-operative emergency laparotomy patients | Paisley | Single | Not stated |
| Jones^36^ | 2016 | NELA | Abstract | Multi-faceted approach to emergency general surgery service modernisation | Nottingham | Single | Positive impact |
| Hussain^37^ | 2016 | NELA | Abstract | Increasing number of surgeons on call to improve patient outcomes of emergency laparotomy | Stoke-on-Trent | Single | Positive impact |
| Seyed-safi^37^ | 2016 | NELA | Abstract | Improving outcomes for NELA patiets aged over 75 | UCLH | Single | Not stated |
| Trotter^37^ | 2016 | NELA | Abstract | Using NELA to effect and demonstrate change | Huddersfield | Single | Positive impact |
| Sundararajan^38^ | 2017 | PICAnet | Abstract | Changing perceptions and outcomes in a paediatric cardiac ECLS program | Leeds | Single | Positive impact |
| Magee^39^ | 2016 | TARN | Abstract | Improving anaesthetic documentation for major trauma | St Mary's London | Single | Positive impact |
| Beane^40^ | 2013 | ICNARC-CMP | Abstract | Surveillance and prevention of ventilator associated pneumonia in a trauma ICU | Royal London Hopsital | Single | Positive impact |
| Eshelby^41^ | 2017 | NHFD | Abstract | Development of a multidisciplinary QI bundle to improve care of hip fracture patients at a DGH | Croydon | Single | Positive impact |
| Shaerf^42^ | 2016 | NHFD | Abstract | Hip fracture QI in a DGH - early surgery is achievable and saves lives | North West London | Single | Positive impact |
| NHFD team^43^ | 2017 | NHFD | Unstructured report | Improving THR rate for hip fracture patients | Royal Preston | Single | Positive impact |
| NHFD team^43^ | 2017 | NHFD | Unstructured report | Improving early mobilisation after surgery for hip fracture patients | Portsmouth | Single | Positive impact |
| Razavi^44^ | 2016 | TARN | Poster | Improving performance of services for patients with open fractures | Manchester | Single | Positive impact |
| Stacey^45^ | 2016 | TARN | Poster | Effectiveness of Helicopter Emergency Medical Service operations | Oxford | Multiple | Not stated |
| Mew^46^ | 2016 | TARN | Poster | Linking TARN data with police data to help target prevention efforts | Dorset | Multiple | Not stated |
| Wafer^47^ | 2016 | TARN | Poster | Appropriateness of level of care for paediatric trauma patients | Manchester | Multiple | Not stated |
| Fernandez^48^ | 2016 | TARN | Poster | New multispecialty service to improve care for patients with open lower limb fractures | Coventry | Single | Positive impact |
| Bates^49^ | 2017 | TARN | Poster | Improving timeliness of imaging in older trauma patients | Northumbria | Single | Positive impact |
| McGuffie^23^ | 2016 | STAG | Unstructured report | Local analysis of data has helped improve time to CT and management of penetrating trauma | McGuffie | Single | Not stated |
| Thomson^23^ | 2016 | STAG | Unstructured report | Local dissemination of data has helped re-instigate trauma teams | Victoria Hospital | Single | Not stated |
| Cooper^23^ | 2016 | STAG | Unstructured report | Better data quality have permitted regional planning, local educaiton and changed documentation | Aberdeen | Multiple | Not stated |
| Dignon^23^ | 2016 | STAG | Unstructured report | Local analyses of data have improved CT scanning processes | Glasgow Royal Infirmary | Single | Not stated |
| Smith^23^ | 2016 | STAG | Unstructured report | Local analysis and dissemination of data have facilitated education and identified a target for QI | QRH Glasgow | Single | Not stated |
| McMahon^23^ | 2016 | STAG | Unstructured report | Data used to create, monitor and improve timelines for trauma patients | Royal Alexandra and Inverclyde | Single | Not stated |
| Wintour^23^ | 2016 | STAG | Unstructured report | Data used to increase numbers of consultants and change pre-hospital and imaging processes | Raigmore | Single | Not stated |
| Key^23^ | 2016 | STAG | Unstructured report | Improved timeliness of CT head scanning | Monklands | Single | Not stated |
| Maclean^23^ | 2016 | STAG | Unstructured report | Improved documentation, guidelines and resuscitation room layout | Wishaw | Single | Not stated |
| Kerslake^23^ | 2016 | STAG | Unstructured report | Improved timeliness of CT scanning and point of care coagulation testing | Edinburgh | Single | Not stated |
| Johnston^23^ | 2016 | STAG | Unstructured report | Improved timeliness of CT head scanning | Ninewells | Single | Not stated |
| NHFD^50^ | 2015 | NHFD | Unstructured report | Early supported discharge | Wirral | Single | Positive impact |
| NHFD^50^ | 2015 | NHFD | Unstructured report | Time to surgery | York | Single | Positive impact |
| NHFD^50^ | 2015 | NHFD | Unstructured report | Intentional rounding to prevent falls | Peterborough | Single | Positive impact |
| NHFD^51^ | 2014 | NHFD | Unstructured report | Improving care for patients with dementia or delerium | Kingston | Single | Not stated |
| NHFD^51^ | 2014 | NHFD | Unstructured report | Service redesign to improve BPT compliance | Warrington | Single | Positive impact |
| NHFD^51^ | 2014 | NHFD | Unstructured report | Appointment of orthogeriatrician and new care pathway to improve BPT achievement | Ealing | Single | Positive impact |
| NHFD^51^ | 2014 | NHFD | Unstructured report | Extra physiotheapy to improve post-op mobilisation | Taunton | Single | Positive impact |
| NHFD^51^ | 2014 | NHFD | Unstructured report | New hip care unit, nutritional policy, nerve block service to improve length of stay | Newcastle | Single | Positive impact |
| NHFD^51^ | 2014 | NHFD | Unstructured report | Embedded PROM collecton within NHFD process | Coventry | Multiple | Not stated |
| NHFD^51^ | 2014 | NHFD | Unstructured report | Appintment of orthogeriatrician, new dedicated hip fracture ward, nerve blocks, pre-op carbohydrate drinks anve improved mortality | Calderdale | Single | Positive impact |
| NHFD^51^ | 2014 | NHFD | Unstructured report | Additional phyio and OT improved LoS | Portsmouth | Single | Positive impact |
| NHFD^51^ | 2014 | NHFD | Unstructured report | Service redesign to reduce time to surgery, improve mortality and BPT attainment. | Chertsey | Single | Positive impact |
| NHFD^51^ | 2014 | NHFD | Unstructured report | Introduction of weekend trauma list to reduce time to surgery | Milton Keynes | Single | Positive impact |
| NHFD^52^ | 2013 | NHFD | Unstructured report | New orthogeriatric service has improve BPT achievement | Airedale | Single | Positive impact |
| NHFD^52^ | 2013 | NHFD | Unstructured report | Increased orthogeriaric service has improved time to surgery and BPT attainment | North Manchester | Single | Positive impact |
| NHFD^52^ | 2013 | NHFD | Unstructured report | Change in pre-op assessment improve time to surgery | Lewisham | Single | Positive impact |
| NHFD^52^ | 2013 | NHFD | Unstructured report | Service redesign has reduced length of stay | James Cook | Single | Positive impact |
| NHFD^52^ | 2013 | NHFD | Unstructured report | Improving and sustaining the rate of pressure ulcers | Royal Liverpool | Single | Positive impact |
| NHFD^52^ | 2013 | NHFD | Unstructured report | Service redesign has reduced time to theatre | Exeter | Single | Positive impact |
| NHFD^52^ | 2013 | NHFD | Unstructured report | Reducing time to surgery | Boston | Single | Positive impact |
| NHFD^52^ | 2013 | NHFD | Unstructured report | Service redesign to improve Best Practice Tariff (BPT) attainment | East Lancashire | Single | Positive impact |
| NHFD^53^ | 2012 | NHFD | Unstructured report | Service redesign to improve BPT attainment | Salisbury | Single | Positive impact |
| NHFD^53^ | 2012 | NHFD | Unstructured report | Better real-time data collectinon and dissemination has facilitated improvements in care to reduce time to surgery | Arrowe Park | Single | Positive impact |
| NHFD^53^ | 2012 | NHFD | Unstructured report | Service redesign has reduced pressure ulcers and length of stay | Russells Hall | Single | Positive impact |
| NHFD^53^ | 2012 | NHFD | Unstructured report | New hip fracture unit has improved pressure ulcers, BPT and mortality | Carshalton | Single | Positive impact |
| NHFD^53^ | 2012 | NHFD | Unstructured report | Change programme has reduced length of stay and mortality | Carmathen | Single | Positive impact |
| NHFD^53^ | 2012 | NHFD | Unstructured report | Service redesign has improved BPT attainment, length of stay and mortality | Pinderfields | Single | Positive impact |
| NHFD^53^ | 2012 | NHFD | Unstructured report | Service redesign reduced mortality. Additional telephone follow-up prviding more comprehensive picture of outcomes | Exeter | Single | Positive impact |
| NHFD^53^ | 2012 | NHFD | Unstructured report | Additional telephone follow-up using NHFD data has demonstrated improvements in post-op mobility | Basingstoke | Single | Positive impact |
| NHFD^53^ | 2012 | NHFD | Unstructured report | Service redesighas reduced time to surgery and length of stay | St Peter's | Single | Positive impact |
| NHFD^53^ | 2012 | NHFD | Unstructured report | Service redesign to improve BPT attainment | Chelsea & Westminster | Single | Positive impact |
| NHFD^53^ | 2012 | NHFD | Unstructured report | Better data collection and dedicated have improved processes and outcomes | Sheffield | Single | Positive impact |
| NHFD^53^ | 2012 | NHFD | Unstructured report | Better MDT collaboration has improved care after hip fracture | Isle of Wight | Single | Positive impact |
| NHFD^53^ | 2012 | NHFD | Unstructured report | QI programme has improved care after hip fracture | Northumbria | Single | Positive impact |
| NHFD^54^ | 2011 | NHFD | Unstructured report | Fracture liaison service improving osteoporosis treatment | Woolwich | Single | Positive impact |
| NHFD^54^ | 2011 | NHFD | Unstructured report | Incremental changes to dedicated service have improved BPT achievement | Dudley | Single | Positive impact |
| NHFD^54^ | 2011 | NHFD | Unstructured report | Care pathway redesign has improved patient satisfaction and reduced time to surgery and length of stay | South Devon | Single | Positive impact |
| NHFD^54^ | 2011 | NHFD | Unstructured report | Monthly scorecard used to to facilitate additional trauma lists which reduced time to surgery | Sutton Coldfield | Single | Positive impact |
| NHFD^54^ | 2011 | NHFD | Unstructured report | Improved data quality used to engage senior clinicians and improved bone scans | Coventry | Single | Not stated |
| NHFD^54^ | 2011 | NHFD | Unstructured report | Appointment of orthogeriatricians has improved perioperative geriatric and bone health assessments | South Tees | Single | Positive impact |
| NHFD^54^ | 2011 | NHFD | Unstructured report | NHFD data used to appoint new orthogeriatrician with subsequent improvements in care | Bristol | Single | Positive impact |
| NHFD^54^ | 2011 | NHFD | Unstructured report | NHFD data used to provide extra services with subsequent improvement in BPT and length of stay | Ipswich | Single | Positive impact |
| NHFD^55^ | 2010 | NHFD | Unstructured report | Data has helped focus minds and improve MDT collaboration to reduce mortality | Gloucestershire | Single | Positive impact |
| NHFD^55^ | 2010 | NHFD | Unstructured report | Pathway reviewed to improve time to surgery, post-op mobilisation and length of stay | James Cook | Single | Positive impact |
| NHFD^55^ | 2010 | NHFD | Unstructured report | A fast track guideline to improve clinical standards and reduce adverse incidents | James Paget Hospital | Single | Positive impact |
| NHFD^55^ | 2010 | NHFD | Unstructured report | New proforma, orthogeriatrcian and dedicated trauma beds being used to reduce delays and pressure ulcers. | Maidstone | Single | Not stated |
| NHFD^55^ | 2010 | NHFD | Unstructured report | MDT trauma group reduced time to surgery and length of stay | Mayday | Single | Positive impact |
| NHFD^55^ | 2010 | NHFD | Unstructured report | New ortho-geriatric unit and MDT steering group reduced delays to surgery | Royal Berkshire | Single | Positive impact |
| NHFD^55^ | 2010 | NHFD | Unstructured report | Collaborative MDT working improved tme to surgery, length of stay and mortality | Royal Surrey | Single | Positive impact |
| NHFD^55^ | 2010 | NHFD | Unstructured report | Training programme and management protocol reduced incidence of pressure ulcers | Salford | Single | Positive impact |
| SICSAG^56^ | 2015 | SICSAG | Unstructured report | Tracheostomy safety project shared through SICSAG network and completed bytrainees using SICSAG data | Ninewells | Multiple | Not stated |
| NELA^57^ | 2016 | NELA | Unstructured report | Improved data quality by engaging with trust quality & safety team | Royal Sussex Hospital | Single | Not stated |
| NELA^57^ | 2016 | NELA | Unstructured report | Improved case ascertainment after clinicians given resources to improve data collection | Worthing | Single | Positive impact |
| NELA^57^ | 2016 | NELA | Unstructured report | New pathway improved risk assessment | Southport | Single | Positive impact |
| NELA^57^ | 2016 | NELA | Unstructured report | New booking system improved risk assessment | King's Lynn | Single | Positive impact |
| NELA^57^ | 2016 | NELA | Unstructured report | New boarding card improved time to surgery | Great Western Hospital | Single | Not stated |
| NELA^57^ | 2016 | NELA | Unstructured report | Surgical leadership has improved consultant presence in theatre | Medway | Single | Positive impact |
| NELA^57^ | 2016 | NELA | Unstructured report | Service reorginsation ahs improved consultant presence in theatre | Royal Preston | Single | Positive impact |
| NELA^57^ | 2016 | NELA | Unstructured report | MDT collaboration as improved rate of eldery care assessment | Kings Mill | Single | Not stated |
| NELA^58^ | 2017 | NELA | Unstructured report | Clinician engagement with data has improved data quality | Birmingham | Single | Not stated |
| NELA^58^ | 2017 | NELA | Unstructured report | Clinician engagement with data has improved time to first consultant surgeon review | University College London Hospital | Single | Not stated |
| NELA^58^ | 2017 | NELA | Unstructured report | Service reorginsation has improved theatre timeliness and efficiency | Salisbury | Single | Not stated |
| NELA^58^ | 2017 | NELA | Unstructured report | Improved risk assessment drove increased consultant presence in theatre | Ysbyty Gwynedd | Single | Not stated |
| NELA^58^ | 2017 | NELA | Unstructured report | Consultant surgeon presence has driven demonstrated improvements | King's College Hospital | Single | Not stated |
| NELA^58^ | 2017 | NELA | Unstructured report | MDT collaboration improved risk assessment, direct consultant supervision, critical care admission and timely CT imaging with subsequent improvement in mortality | Kingston | Single | Positive impact |
| NELA^58^ | 2017 | NELA | Unstructured report | New booking form and weekly reminders improved risk assessment | Southampton | Single | Positive impact |
| NELA^58^ | 2017 | NELA | Unstructured report | Departure of key staff required re-establishment of MDT improvement team to regain previous performance levels and sustain improvement | Bath | Single | Not stated |
| Bannon^59^ | 2017 | NLCA | Unstructured report | New data validation processes have improved data quality | St Helens | Single | Not stated |
| Shackloth^60^ | 2017 | NLCA | Unstructured report | Reflections on outliying - how to check data | Liverpool | Single | Not stated |
| Walton^61^ | 2019 | NHFD | QI report | Retrospective case series comparing outcomes after service reconfiguration | Brighton and Sussex University Hospitals | Single | Positive impact |
| Perkins^62^ | 2014 | TARN | QI report | Targeted improvement programme in a major trauma centre | Royal London Hopsital | Single | Positive impact |
| Vanhegan^63^ | 2019 | NJR | Evaluation of QI project | Improving compliance with best practice tariffs | Chelsea and Westminster Hopsital NHS Foundation Trust | Single | Positive impact |
| Mc Williams^64^ | 2019 | ICNARC-CMP | Evaluation of QI project | Improving early postoperative mobilisation | Birmingham | Single | Mixed impacts |
| Frawley^65^ | 2019 | ICNARC-CMP | Evaluation of QI project | Introducing new sedation policy in ICU | Newport | Single | Positive impact |
| Aggarwal^66^ | 2019 | NELA | Evaluation of QI project | Using an evidence based bundle in the Emergency Laparotomy Collaborative | South of England (28 hospitals) | Multiple | Positive impact |
| Bollard^67^ | 2017 | NLCA | Abstract | Pre-habilitation for lung cancer surgery patients | London | Single | Not stated |
| Pegba-Otemolu^68^ | 2019 | NELA | Abstract | improving data entry completion for NELA | Oxford | Single | Positive impact |
| Doyle^69^ | 2019 | NELA | Evaluation of QI project | Implementation of a QI bundle improves survival but not kidney injury | 4 hospitals in England | Multiple | No impact |
| Peden^70^ | 2019 | NELA | Evaluation of QI project | National QI programme to improve survival after emergency abdominal surgery | England | Multiple | Mixed impacts |
| Ilyas^71^ | 2019 | NELA | Abstract | Local analysis of NELA data to identify sepsis | St James University Hospital | Single | Not stated |
| Bridgestock^72^ | 2019 | NELA | Poster | Improving risk assessment documentation early mobilisation after emergency laparotomy | Queen Elizabeth University Hospital Glasgow | Single | Positive impact |
| Hawkes^73^ | 2018 | NHFD | Commentary / editorial | Improving care of hip fractures | Banbury | Single | Positive impact |
| Bryce^74^ | 2019 | TARN | Abstract | Involving students to improve data collection | Truro | Single | Positive impact |
| De Las Casas^75^ | 2019 | NELA | Abstract | Improvements in perioperative services for older people | Dartford and Gravesham NHS Trust | Single | Positive impact |
| Findley^75^ | 2019 | NELA | Abstract | Introducing postoperative analgesia | Ipswich Hospital, East Suffolk, North Essex Foundation Trust | Single | Positive impact |
| Verschueren^76^ | 2019 | NELA | Abstract | Improved documentation of predicted preoperative risk | University College London Hospital | Single | Positive impact |
| Liverpool Research Trainee Collaborative^77^ | 2019 | NELA | Poster | Improving data ascertainement and real time data analysis | Liverpool | Single | Positive impact |
| Reeds^78^ | 2019 | NELA | Poster | Improving adherence to stndards for consultants | Whipps Cross University Hospital | Single | Positive impact |
| Eckersley^79^ | 2019 | NHFD | Abstract | Increasing physiotherapy access for hip fracture patients | Manchester | Single | Positive impact |
| Jones^75^ | 2019 | NELA | Abstract | Improving data collection with a mobile application | Liverpool | Single | Positive impact |
| Paul^80^ | 2019 | PQIP | Unstructured report | Alleviating postoperative thirst | Brighton and Sussex University Hospital | Single | Positive impact |
| Satur^81^ | 2019 | ACS | Unstructured report | Reducing waits for urgent cardiac bypass surgery | University Hospital of North Midlands | Single | Positive impact |
| Lodge^59^ | 2018 | NLCA | Unstructured report | Improvement in all areas of NLCA | Portsmouth Hospital | Single | Positive impact |
| National Prostate Cancer Audit^82^ | 2019 | NPCA | Unstructured report | Reducing genito-urinary complications | Gloucestershire Hospitals NHS Foundation Trust | Single | Positive impact |
| Egginton^83^ | 2019 | SSISS | Unstructured report | Using a multidisciplinary approach to reduce the incidence of SSIs | Sheffield | Single | Positive impact |
| Oliver^75^ | 2019 | NELA | Abstract | Improving pain management | Cardiff | Single | Positive impact |
| Trainer^75^ | 2019 | NELA | Abstract | Improving geriatric review after emergency laparotomy | London | Single | Positive impact |
| Gladstone^76^ | 2018 | NELA | Abstract | Improving risk scoring with electronic booking | Bedford | Single | Mixed impacts |
| Walker^80^ | 2019 | PQIP | QI report | Reducing postoperative thirst | London | Single | Not stated |
| Ribeiro^80^ | 2019 | PQIP | Poster | Improving pre-operative anaemia | Colchester | Single | Positive impact |
| Farrant^84^ | 2019 | PQIP | Poster | Enhanced Recovery for major GI surgery | York | Single | Not stated |
| Chirvasuta^85^ | 2019 | PQIP | Poster | Individualised risk assessment for colorectal surgery | York | Single | Positive impact |
| Chiwera^83^ | 2019 | SSISS | QI report | Improving surgical wound documentation to reduce infection | London | Single | Not stated |
| George^86^ | 2018 | SSISS | QI report | Reducing infection in hip fracture patients | Chertsey | Single | Positive impact |
| Donnelly^87^ | 2019 | STAG | QI report | Improving time to CT scanning | Hairmyres | Single | Not stated |
| Chohan^88^ | 2018 | SICSAG | QI report | Improving early mobilisation in ICU | Monklands | Single | Positive impact |
| Saunders^89^ | 2018 | NELA | Unstructured report | NELA feedback forms | Royal Victoria, Newcastle | Single | Not stated |
| Satisha^89^ | 2018 | NELA | Unstructured report | MDT review meetings | Darent Valley | Single | Positive impact |
| Riley^89^ | 2018 | NELA | Unstructured report | Improving access to elderly care liaison | Salford | Single | Not stated |
| Kerslake^90^ | 2018 | STAG | Unstructured report | Implementation of a tiered trauma team response | Edinburgh | Single | Not stated |
| Rae^91^ | 2018 | SHFA | Unstructured report | Early mobilisation after hip fracture | Lanarkshire | Single | Positive impact |
| SICSAG^92^ | 2018 | SICSAG | Unstructured report | Managing Pain in Intensive Care | Raigmore | Single | Positive impact |

1. Aggarwal G, Quiney N. Emergency laparotomy, the ELC and quality improvement. *Bull R Coll Surg Engl*. 2017;99(7):278-280.

2. Gousia K, King A, Nwulu U, Coulton S, Peckham S. Interim report for the evaluation of the Emergency Laparotomy Collaborative project. 2016;(August).

3. Aveling E, Martin G, Herbert G, Armstrong N. Comparative case studies of the clinical community model in practice. *Soc Sci Med*. 2017;173.

4. Russell GK, Jimenez S, Martin L, et al. A multicentre randomised controlled trial of reciprocal lung cancer peer review and supported quality improvement: results from the improving lung cancer outcomes project. *Br J Cancer*. 2014;110(8):1936-1942.

5. Mayor S. Lung cancer teams pair up to review care processes and improve outcomes. *BMJ*. 2012;344:e2770.

6. The Royal College of Physicians. Improving care for lung cancer patients : a collaborative approach. Improvement stories from lung cancer teams. 2012.

7. Armstrong N, Martin G, Willars J, Shaw E, Dixon-woods M. Evaluation of Participation in the Improving Lung Cancer Outcomes Project ( ILCOP ). 2012;(April):1-34.

8. Aveling E et al. Reciprocal peer review for quality improvement: an ethnographic case study of the Improving Lung Cancer Outcomes Project. *BMJ Qual Saf*. 2012.

9. Armstrong N, Herbert G, Aveling EL, Dixon-Woods M, Martin G. Optimizing patient involvement in quality improvement. *Heal Expect*. 2013;16(3):36-47.

10. Pearse R. Enhanced peri-operative care for high-risk patients (EPOCH) trial: a stepped wedge cluster randomised trial of a quality improvement intervention for patients undergoing emergency laparotomy. *Pragmatic Clin Trials Unit Queen Mary Univ London*. 2014;(April):1-28.

11. Basser MR. *PROMS Benefits Case Study: Patient Reported Outcome Measures (PROMs) Outputs*.; 2015.

12. Partridge T et al. Improving patient reported outcome measures (PROMs) in total knee replacement by changing implant and preserving the infrapatella fatpad: a quality improvement project. *BMJ Qual Improv reports*. 2016;5(1).

13. Graham S, Dahill M, Robinson D. Reducing returns to theatre for neck of femur fracture patients. *BMJ Qual Improv Reports*. 2017;6(1):u215756.w6261.

14. R. Lisk KY. Reducing mortality from hip fractures: a systematic quality improvement programme. *BMJ Qual Improv Reports*. 2014;3(1):u205006.w2103-u205006.

15. Chamberlain M, Pugh H. Improving inpatient care with the intriduction of a hip fracture pathway. *BMJ Qual Improv reports*. 2015:2015-2018.

16. Gupta A. The effectiveness of geriatrician-led comprehensive hip fracture collaborative care in a new acute hip unit based in a general hospital setting in the UK. *J R Coll Physicians Edinb*. 2014;44(1):20-26.

17. NHFD. *QI Examples - Report Vignettes and Good News Stories. Unpublished.*

18. D. H, J. B, C. B, et al. Improving the care of patients with a hip fracture: a quality improvement report. *BMJ Qual Saf*. 2015;24(8):532-538.

19. Middleton M, Wan B, da Assunçāo R. Improving hip fracture outcomes with integrated orthogeriatric care: a comparison between two accepted orthogeriatric models. *Age Ageing*. 2016;46(3):465-470.

20. Howell SJ. Abdominal aortic aneurysm repair in the United Kingdom: an exemplar for the role of anaesthetists in perioperative medicine. *Br J Anaesth*. 2017;119(suppl_1):i15-i22.

21. Taylor A, Neuburger J, Walker K, Cromwell D, Groene O. How is feedback from national clinical audits used? Views from English National Health Service trust audit leads. *J Health Serv Res Policy*. 2016;21(2):91-100.

22. Davenport RA et al. A major trauma centre is a specialty hospital not a hospital of specialties. *Br J Surg*. 2010;97(1):109-117.

23. Scottish Trauma Audit Group. *Audit of Trauma Management in Scotland . Annual Report*.; 2016.

24. National Services Scotland. *Standards of Care for People with Hip Fractures. Spotlight on: Elderly Care Orthopaedic Nurses*.; 2017.

25. National Services Scotland. Standards of Care for people with hip fractures. Spotlight on: Patients’ return to original place of residence within 30 days. *Framework*. 2017:57-58.

26. Scottish Hip Fracture Audit. *Standards of Care for People with Hip Fractures. Spotlight on: Fascia Iliaca Blocks*.; 2017.

27. Jimenez S, Martin L, Aveling E, Martin G, Woolhouse I. The improving lung cancer outcomes project: a study of the feasibility of a national reciprocal peer review and facilitated quality improvement programme. *Thorax*. 2011;66(Suppl 4):P154.

28. Patel PK, Tezas S, Shams M, Choudhury A, Sgourikas G. Ongoing Quality improvement through NELA in Year 2 in a District General Hospital. *Br J Surg*. 2017;104(S6, SI):161.

29. Shilston J, Godsiff D. Enhancing care for our high-risk laparotomy patients at Stoke Mandeville Hospital. *Anaesthesia*. 2016;71:35.

30. Kersan L, Young D. Emergency laparotomy: A Scottish perspective. *Anaesthesia*. 2016;71:28.

31. McDermott FD, Lyons NJ, Noble EJ, McCormick BA, Bethune R. A quality improvement project on pre-operative risk stratification for emergency laparotomies using NELA data. *Br J Surg*. 2016;103(6, SI):119.

32. AAGBI. Abstracts of the AAGBI WSM London, UK, 11-13 Jan 2017. *Anaesthesia*. 2017;72(2, SI):90.

33. Cartwright C, Griffiths M. Risk stratification and postoperative destination in a district general hospital. *Anaesthesia*. 2015;70:24.

34. Balock S, Charters S, Murthy G, Mccann J, P. N. Has one year of National Emergency Laparotomy Audit changed the quality of care for patients? *Anaesthesia*. 2015;70:91.

35. AAGBI. Abstracts of the AAGBI GAT Annual Scientific Meeting 2014. *Anaesthesia*. 2014;69.

36. ASGBI. ASGBI abstracts 2016. *Br J Surg*. 2016;103:104-209.

37. ASGBI. ASGBI abstracts 2017 - NELA prize. *Br J Surg*. 2017;104:83-243.

38. Sundararajan S, Lumsden J, Bentham J. Changing perceptions and changing outcomes in a paediatric cardiac ECLS program. *Eur J Heart Fail*. 2017;19:24-25.

39. Magee D, Wickham A, Monk A. Anaesthetic documentation for major trauma in a major trauma centre. *Anaesthesia*. 2016;71:42.

40. Beane A, Hill L, Hadley J, Millar M. Surveillance strategy and prevention of ventilator associated pneumonia in a trauma ICU. *Intensive Care Med*. 2013;39:S314.

41. Eshelby S, Saxena S, Oliver M. Development of a multidisciplinary quality improvement bundle to improve perioperative care in the management of neck of femur fractures at a district general hospital. *Anaesthesia*. 2017;72:35.

42. D. S, S. M. Hip fracture quality improvement in a district general hospital - Early surgery is achievable and saves lives. *Int J Surg*. 2016;36(Supplement 1):S108.

43. Royal College of Physicians. *National Hip Fracture Database (NHFD) Annual Report 2017*.; 2017.

44. Razavi L, Trompeter A. *TARN Improvement Award. Open Fracture Performance.*; 2016.

45. Stacey L, Ward M, Frobisher P. *TARN Improvement Award. Effectiveness of HEMS Operations.*; 2016.

46. Mew I, Governance C, Medical T, Officer S. *TARN Improvement Awards. Motorcycle Road Deaths and Serious Injury.*

47. Wafer M. *TARN Improvement Awards. Appropriateness of Level of Care.*; 2016.

48. Fernandez M. *TARN Improvement Awards. Open Lower-Limb Fracture Performance.*; 2016.

49. Bates C, Em N, Trauma E. *TARN Imporvement Awards. Improvements in Care.*; 2017.

50. Royal College of Physicians. *National Hip Fracture Database (NHFD) Annual Report 2015*.; 2015.

51. Royal College of Physicians. *National Hip Fracture Database (NHFD) - Extended Report 2014*.; 2014.

52. Johansen A, Wakeman R, Boulton C, Plant F, Roberts J, Williams A. *National Hip Fracture Database - National Report 2013*.; 2013.

53. Currie C, Partridge M, Plant F, Roberts J. The National Hip Fracture Database National Report 2012. 2012:1-115.

54. Currie C, Partridge M, Plant F, Roberts J. *The National Hip Fracture Database National Report 2011*.; 2011.

55. Currie C, Fleming S, Plant F, Wakeman R. *The National Hip Fracture Database National Report 2010*.; 2010.

56. Scottish Intensive Care Society Audit Group. Quality Improvement examples. https://www.hrsa.gov/quality/toolbox/508pdfs/qualityimprovement.pdf. Published 2011.

57. NELA Project Team. The second patient report of the National Emergency Laparotomy Audit. 2016;(July):1-160.

58. NELA Project Team. *The Third Patient Report of the National Emergency Laparotomy Audit ( NELA )*.; 2017.

59. National Lung Cancer Audit. *National Lung Cancer Audit Annual Report 2017*.

60. Shackloth M. Reflections on outlying. *NLCA Qual Improv Work*. 2017.

61. Walton TJ, Bellringer SF, Edmondson M, Stott P, Rogers BA. Does a dedicated hip fracture unit improve clinical outcomes? A five-year case series. *Ann R Coll Surg Engl*. 2019;101(3):215-519.

62. Perkins ZB, Maytham GD, Koers L, Bates P, Brohi K, Tai NRM. Performance improvement programmes and their effect on patient outcome. Impact on outcome of a targeted performance improvement programme in haemodynamically unstable patients with a pelvic fracture. *J Bone Jt Surg*. 2014(96-B):1090-1097.

63. Vanhegan I, Sankey A, Radford W, Ball S, Gibbons C. Trust compliance with best practice tariff criteria for total hip and knee replacement. *Br J Hosp Med (Lond)*. 2019;80(9):537-540.

64. McWilliams D, Snelson C, Goddard H, Attwood B. Introducing early and structured rehabilitation in critical care: A quality improvement project. *Intensive Crit Care Nurs*. 2019;53:79-83.

65. Frawley A, Hickey J, Weaver C, Williams J, Szakmany T. Introducing a new sedation policy in a large district general hospital: before and after cohort analysis. *Anaesthesiol Intensive Ther*. 2019;51(1):4-10.

66. Aggarwal G, Peden CJ, Mohammed MA, et al. Evaluation of the Collaborative Use of an Evidence-Based Care Bundle in Emergency Laparotomy. *JAMA Surg*. 2019;154(5):e190145.

67. Bollard K, Lau K, De Luca B, Hornby C, Ricketts WM. 153: Experiences of setting up a pre-operative optimisation ‘pre-hab’service for patients being considered for lung cancer surgery. *Lung Cancer*. 2017;103:S69.

68. Pegba-Otemolu I, Menon N, Muthusami A, Gilmour J. Evaluation of Performance with Data Entry in the National Emergency Laparotomy Audit: A Tertiary Level Hospital Quality Improvement Project. *Br J Surg*. 2019;106(6, SI):54.

69. Doyle JF, Sarnowski A, Saadat F, et al. Does the Implementation of a Quality Improvement Care Bundle Reduce the Incidence of Acute Kidney Injury in Patients Undergoing Emergency Laparotomy?. *J Clin Med*. 2019;8(8).

70. Peden CJ, Stephens T, Martin G, et al. Effectiveness of a national quality improvement programme to improve survival after emergency abdominal surgery (EPOCH): a stepped-wedge cluster-randomised trial. *Lancet*. 2019;0(0).

71. Ilyas C, Fearns E, Varley K, Schofield C, Sivanandan I. Utilising process mapping to improve laparotomy care in the emergency department: a collaborative NELA QI project. *Anaesthesia*. 2019;74(3, SI):80.

72. Bridgestock C, Pickering S, Witherspoon MP. ELLSA@ QEUH: Improving Patient Outcomes After Emergency Abdominal Surgery. In: *ANAESTHESIA*. Vol 74. ; 2019:31.

73. Hawkes N. The BMJ Awards 2018: Patient Safety Team of the Year. *Bmj*. 2018;361:k1699.

74. Bryce S, Cheema K., Warrick B., Mortiz G. Truro Trauma Scribes: Students as scribes to improve trauma documentation and educational experience. *Trauma (United Kingdom)*. 2019;21(3):229.

75. Abstracts of WSM 2019, 9-11 January 2019, London, UK. *Anaesthesia*. 2019;74:9-79.

76. Abstracts of the AAGBI WSM London, London, UK, 10-12 January 2018. *Anaesthesia*. 2018;73:9-79.

77. Liverpool Trainee Research Collaborative, Merseyside Anaesthetic Group for Improving Quality (MAGIQ). The use of an anonymised phone app improves NELA ascertainment and allows real time data analysis. 2016:3.

78. Reeds MG, Thunga S, Andreani SM, Taylor FGM. Are We Meeting The National Emergency Laparotomy Audit ( NELA ) Consultant Standards ? 94(Asa 4):94.

79. Eckersley P, Heneghan J, Barton O. A project to improve physiotherapy performance in relation to measures included in The National Hip Fracture Database annual reports...The Chartered Society of Physiotherapy UK Conference 2018, Birmingham, UK, 19-20 October 2018. *Physiotherapy*. 2019;105:e59-e60.

80. PQIP. PQIP ANNUAL REPORT 2018-19. 2018.

81. NICOR. National Adult Cardiac Surgery Audit Report. 2019:1-44.

82. NPCA. NPCA Quality Improvement case studies. 2018:0-1.

83. Health Protection Agency. Surveillance of Surgical Site Infections in NHS Hospitals in England. 2019.

84. Farrant M, Trainee F, Walkington J, Ygh CA. Enhanced Recovery after Colorectal Surgery at York Hospital. *PQIP Abstr Compet*. 2019;(2014):2014.

85. Chirvasuta R, Walkington J. Individualised Risk Assessment for Colorectal Surgery. *PQIP Abstr Compet*. 2019;84(April):2019.

86. Public Health England. Surveillance of surgical site infections in NHS hospitals in England, 2017 to 2018. 2018;(December):1-51.

87. Scotish Trauma Audit Group. STAG annual report 2019 - Shared Learning. 2019;21.

88. Chohan S, Ash S, Senior L. A team approach to the introduction of safe early mobilisation in an adult critical care unit. *BMJ Open Qual*. 2018;7(4):e000339.

89. NELA Project Team. Fourth Patient Report of the National Emergency Laparotomy Audit. *RCoA London*. 2018;(November):1-137.

90. Quality improvement for patients with trauma – responses from STAG Clinical Leads. 2017:2017.

91. Rae C. Scottish Standards of Care for People with Hip Fractures Autumn Spotlight on ...... Physiotherapy and Occupational Therapy. 2017:8-10.

92. Sicsag. *Audit of Critical Care in Scotland*.; 2018.

### Table S5: Publishing Characteristics of Reports

| **Manuscript type** | **Count** | **%** |
| --- | --- | --- |
| Unstructured report | 120 | 57 |
| Abstract | 42 | 21 |
| Poster | 14 | 7 |
| QI report (peer-reviewed) | 14 | 7 |
| Evaluation of QI project (peer-reviewed) | 11 | 5 |
| Commentary / editorial | 6 | 3 |
| Protocol | 1 | 0 |
| Secondary analysis of empirical data | 1 | 0 |
| **Totals** | **209** | **100** |
|  |  |  |
| **Target Audience** | **Count** | **%** |
| Audit specific | 134 | 64 |
| Anaesthesia | 34 | 16 |
| Surgery | 18 | 9 |
| QI / HSR | 8 | 4 |
| Intensive care | 6 | 3 |
| General clinical | 4 | 2 |
| Physicians | 4 | 2 |
| Nursing | 1 | 0 |
| Management | 0 | 0 |
| **Totals** | **203** | **100** |

### Table S6: Types of quality indicators used (all reports)

| **Type of Indicator** | **No. of reports** | **%** |
| --- | --- | --- |
| Structure | 3 | 1 |
| Process | 144 | 69 |
| Outcome | 94 | 45 |
| Not stated | 16 | 8 |

### Table S7: The purposes of and activities involving the use of NCA data (all reports)

| **Purpose of using NCA data** | **No. of reports** | **%** |
| --- | --- | --- |
| Identify target for QI | 116 | 55 |
| Prioritise between QI projects | 4 | 2 |
| Initiate a QI project | 73 | 35 |
| Monitor ongoing QI project | 151 | 72 |
| Other | 18 | 8 |
| Not stated | 8 | 4 |
|  |  |  |
| **Activities using NCA data** | **No. of reports** | **%** |
| Collection of additional data using NCA criteria | 30 | 14 |
| Local analysis of NCA data | 166 | 79 |
| Local dissemination of NCA data | 49 | 23 |
| Other | 21 | 10 |
| Not stated | 13 | 6 |

### Table S8: Reported Impact(s) of QI Projects

| **Impact** | **No of reports** | **%** |
| --- | --- | --- |
| Positive impact | 128 | 61 |
| Mixed impact(s) | 4 | 2 |
| No impact | 4 | 2 |
| Negative impact | 1 | 0 |
| Not stated | 72 | 34 |
| **Totals** | **209** | **100** |

### Table S9: Quality Assessment of Peer-Reviewed QI Reports

|  |  | **Adequately described** | |
| --- | --- | --- | --- |
|  |  | **No. of Reports** | **Percentage of reports** |
| **Title** | Indicate that the manuscript concerns an initiative to improve healthcare | 25 | 100 |
| **Abstract** | a. Provide adequate information to aid in searching and indexing | 25 | 100 |
|  | b. Summarize all key information from various sections of the text using the abstract format of the intended publication or a structured summary such as: background, local problem, methods, interventions, results, conclusions | 25 | 100 |
| **INTRODUCTION** | **WHY DID YOU START?** |  |  |
| **Problem Description** | Nature and significance of the local problem | 24 | 96 |
| **Available knowledge** | Summary of what is currently known about the problem, including relevant previous studies | 21 | 84 |
| **Rationale** | Informal or formal frameworks, models, concepts, and/or theories used to explain the problem, any reasons or assumptions that were used to develop the intervention(s), and reasons why the intervention(s) was expected to work | 25 | 100 |
| **Specific aims** | Purpose of the project and of this report | 25 | 100 |
| **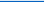**   \| **METHODS** \| \| --- \| | **WHAT DID YOU DO?** |  |  |
| **Context** | Contextual elements considered important at the outset of introducing the interventions | 18 | 72 |
| **Interventions** | a. Description of the intervention(s) in sufficient detail that others could reproduce it | 20 | 80 |
|  | b. Specifics of the team involved in the work | 17 | 68 |
| **Study of the Intervention(s)** | a. Approach chosen for assessing the impact of the intervention(s) | 20 | 80 |
|  | b. Approach used to establish whether the observed outcomes were due to the intervention(s) | 17 | 68 |
| **Measures** | a. Measures chosen for studying processes and outcomes of the intervention(s), including rationale for choosing them, their operational definitions, and their validity and reliability | 20 | 80 |
|  | b. Description of the approach to the ongoing assessment of contextual elements that contributed to the success, failure, efficiency, and cost | 10 | 40 |
|  | c. Methods employed for assessing completeness and accuracy of data | 8 | 32 |
| **Analysis** | a. Qualitative and quantitative methods used to draw inferences from the data | 19 | 76 |
|  | b. Methods for understanding variation within the data, including the effects of time as a variable | 10 | 40 |
| **Ethical Considerations** | Ethical aspects of implementing and studying the intervention(s) and how they were addressed, including, but not limited to, formal ethics review and potential conflict(s) of interest | 10 | 40 |
| **RESULTS** | **WHAT DID YOU FIND?** |  |  |
| **Results** | a. Initial steps of the intervention(s) and their evolution over time (e.g., time-line diagram, flow chart, or table), including modifications made to the intervention during the project | 19 | 76 |
|  | b. Details of the process measures and outcome | 21 | 84 |
|  | c. Contextual elements that interacted with the intervention(s) | 5 | 20 |
|  | d. Observed associations between outcomes, interventions, and relevant contextual elements | 16 | 64 |
|  | e. Unintended consequences such as unexpected benefits, problems,  failures, or costs associated with the intervention(s). | 5 | 20 |
|  | f. Details about missing data | 6 | 24 |
| **DISCUSSION** | **WHAT DOES IT MEAN?** |  |  |
| **Summary** | a. Key findings, including relevance to the rationale and specific aims | 23 | 92 |
|  | b. Particular strengths of the project | 17 | 68 |
| **Interpretation** | a. Nature of the association between the intervention(s) and the outcomes | 21 | 84 |
|  | b. Comparison of results with findings from other publications | 13 | 52 |
|  | c. Impact of the project on people and systems | 19 | 76 |
|  | d. Reasons for any differences between observed and anticipated outcomes, including the influence of context | 14 | 56 |
|  | e. Costs and strategic trade-offs, including opportunity costs | 5 | 20 |
| **Limitations** | a. Limits to the generalizability of the work | 10 | 40 |
|  | b. Factors that might have limited internal validity such as confounding, bias, or imprecision in the design, methods, measurement, or analysis | 14 | 56 |
|  | c. Efforts made to minimize and adjust for limitations | 8 | 32 |
| **Conclusions** | a. Usefulness of the work | 22 | 88 |
|  | b. Sustainability | 15 | 60 |
|  | c. Potential for spread to other contexts | 14 | 56 |
|  | d. Implications for practice and for further study in the field | 17 | 68 |
|  | e. Suggested next steps | 6 | 24 |
| **OTHER INFORMATION** |  |  |  |
| **Funding** | Sources of funding that supported this work. Role, if any, of the funding organization in the design, implementation, interpretation, and reporting | 9 | 36 |
